# Unveiling the therapeutic potential of exogenous β-hydroxybutyrate for chronic colitis in rats: novel insights on autophagy, apoptosis, and pyroptosis

**DOI:** 10.3389/fphar.2023.1239025

**Published:** 2023-09-28

**Authors:** Rasha Abdelhady, Sameh Saber, Mustafa Ahmed Abdel-Reheim, Mohannad Mohammad S. Alamri, Jaber Alfaifi, Masoud I. E. Adam, Lobna A. Saleh, Azza I. Farag, Elsayed A. Elmorsy, Hend S. El-Wakeel, Ahmed S. Doghish, Mohamed E. Shaker, Sara H. Hazem, Heba A. Ramadan, Rabab S. Hamad, Osama A. Mohammed

**Affiliations:** ^1^ Pharmacology and Toxicology Department, Faculty of Pharmacy, Fayoum University, Fayoum, Egypt; ^2^ Department of Pharmacology, Faculty of Pharmacy, Delta University for Science and Technology, Gamasa, Egypt; ^3^ Department of Pharmaceutical Sciences, College of Pharmacy, Shaqra University, Shaqra, Saudi Arabia; ^4^ Department of Pharmacology and Toxicology, Faculty of Pharmacy, Beni-Suef University, Beni Suef, Egypt; ^5^ Department of Family Medicine, College of Medicine, University of Bisha, Bisha, Saudi Arabia; ^6^ Department of Child Health, College of Medicine, University of Bisha, Bisha, Saudi Arabia; ^7^ Department of Medical Education and Internal Medicine, College of Medicine, University of Bisha, Bisha, Saudi Arabia; ^8^ Department of Clinical Pharmacology, Faculty of Medicine, Ain Shams University, Cairo, Egypt; ^9^ Department of Pharmacology and Toxicology, Collage of Pharmacy, Taif University, Taif, Saudi Arabia; ^10^ Department of Human Anatomy and Embryology, Faculty of Medicine, Zagazig University, Zagazig, Egypt; ^11^ Department of Pharmacology and Therapeutics, Qassim College of Medicine, Qassim University, Buraydah, Saudi Arabia; ^12^ Department of Clinical Pharmacology, Faculty of Medicine, Mansoura University, Mansoura, Egypt; ^13^ Physiology Department, Benha Faculty of Medicine, Benha University, Banha, Egypt; ^14^ Physiology Department, Al-baha Faculty of Medicine, Al-baha University, Al-Baha, Saudi Arabia; ^15^ Department of Biochemistry, Faculty of Pharmacy, Badr University in Cairo (BUC), Cairo, Egypt; ^16^ Department of Biochemistry and Molecular Biology, Faculty of Pharmacy (Boys), Al-Azhar University, Cairo, Egypt; ^17^ Department of Pharmacology, College of Pharmacy, Jouf University, Sakaka, Saudi Arabia; ^18^ Department of Pharmacology and Toxicology, Faculty of Pharmacy, Mansoura University, Mansoura, Egypt; ^19^ Department of Microbiology and Immunology, Faculty of Pharmacy, Delta University for Science and Technology, Al Mansurah, Egypt; ^20^ Biological Sciences Department, College of Science, King Faisal University, Al Ahsa, Saudi Arabia; ^21^ Central Laboratory, Theodor Bilharz Research Institute, Giza, Egypt; ^22^ Department of Clinical Pharmacology, College of Medicine, University of Bisha, Bisha, Saudi Arabia

**Keywords:** ulcerative colitis, β-hydroxybutyrate, NLRP3 inflammasome, apoptosis, pyroptosis

## Abstract

Ulcerative colitis (UC) is a chronic relapsing inflammatory disease of the colorectal area that demonstrates a dramatically increasing incidence worldwide. This study provides novel insights into the capacity of the exogenous β-hydroxybutyrate and ketogenic diet (KD) consumption to alleviate dextran sodium sulfate (DSS)-induced UC in rats. Remarkably, both interventions attenuated disease activity and colon weight-to-length ratio, and improved macro and microstructures of the damaged colon. Importantly, both β-hydroxybutyrate and KD curbed the DSS-induced aberrant NLRP3 inflammasome activation as observed in mRNA and protein expression analysis. Additionally, inhibition of the NLRP3/NGSDMD-mediated pyroptosis was detected in response to both regimens. In parallel, these modalities attenuated caspase-1 and its associated consequences of IL-1β and IL-18 overproduction. They also mitigated apoptosis as indicated by the inactivation of caspase-3. The anti-inflammatory effects of BHB and KD were confirmed by the reported decline in the levels of inflammatory markers including MPO, NFκB, IL-6, and TNF-α. Moreover, these interventions exhibited antioxidative properties by reducing ROS production and improving antioxidative enzymes. Their effectiveness in mitigating UC was also evident in the renovation of normal intestinal epithelial barrier function, as shown by correcting the discrepancies in the levels of tight junction proteins ZO-1, OCLN, and CLDN5. Furthermore, their effects on the intestinal microbiota homeostasis were investigated. In terms of autophagy, exogenous β-hydroxybutyrate upregulated BECN-1 and downregulated p62, which may account for its superiority over KD in attenuating colonic damage. In conclusion, this study provides experimental evidence supporting the potential therapeutic use of β-hydroxybutyrate or β-hydroxybutyrate-boosting regimens in UC.

## 1 Introduction

Ulcerative colitis (UC) is a chronic idiopathic inflammatory disorder that affects the mucosal and submucosal lining of the gastrointestinal tract (GIT) generally and the colon specifically (Zohny et al., 2022a; Cavalu et al., 2022). UC and Crohn’s disease constitute the subtypes of inflammatory bowel disease (IBD). Notably, UC is a multifactorial disease underlined by the interaction between a plethora of factors including environmental factors, abnormal immune response, changes in intestinal microbiome as well as genetic predisposition. The clinical presentation of UC comprises abdominal algesia, diarrhea, rectal bleeding, and eventually weight loss (Olén et al., 2020).

Chronic intestinal inflammation is associated with massive leukocyte infiltration. This immune evasion is associated with oxidative stress that is characterized by increased reactive oxygen species (ROS) production. These chemically reactive byproducts significantly contribute to mucosal injury and the impairment of intestinal barrier function (Bhattacharyya et al., 2014). Moreover, another theory stated that the intestinal microbiome plays a principal role in UC pathogenesis where microbial imbalance leads to abnormal immune response (Shreiner et al., 2015). Notably, gut microbiota represents both a remarkable disease biomarker and a therapeutic target (Hajjo et al., 2022).

UC remains to be a global health challenge for a variety of reasons. Firstly, no curative treatment was yet discovered. Secondly, UC can lead to serious life-threatening conditions among which colitis-associated colorectal malignancy is the most important and the most frequent where the risk increases with prolonged UC chronicity (Saber et al., 2020; Abdelhamid et al., 2022b; Nasr et al., 2022). Other potential complications include toxic megacolon and colonic wall erosions. Current therapeutic modalities of UC comprise a range of anti-inflammatory agents such as aminosalicylates, sulfasalazine, and glucocorticoids (Jeong et al., 2019) where their use was limited by 40% remission rate as well as their adverse effects (Antonelli et al., 2018). Additionally, immunosuppressants including thiopurine (azathioprine) and/or anti-TNF-α (Infliximab) were used for the management of refractory and severe cases (Oussalah et al., 2010). Notably, the development of novel therapies for the management of UC is an urgent medical need. The high rate of remission in conjunction with the significant toxicity of current therapeutic approaches makes the management of this illness challenging, primarily because of its multifactorial nature.

Ketogenic diet (KD) establishes a novel dieto-therapeutic approach that was proven beneficial in modulating a variety of health disorders. KD could be defined as forcing the body to use fat as a source of energy instead of carbohydrate leading to induction or achievement of physiologic ketosis. Ketosis is a metabolic state characterized by high blood ketone levels (0.5–3 mmol/L). The state of ketosis can be achieved through a ketogenic diet, which involves restricting carbohydrate intake to very low levels while consuming moderate amounts of protein. In exchange, there is an increased emphasis on fat intake, which should account for approximately 70%–90% of the total caloric intake in the diet (Cahill, 2006).

β-hydroxybutyrate (BHB) is the most abundant ketone compound and it’s produced by the human body during the state of ketosis owing to glucose depletion through β-oxidation of fatty acids by hepatocytes. Notably, it constitutes approximately 75% of total circulating ketone bodies (Puchalska and Crawford, 2017). Moreover, accumulating evidence highlighted the beneficial role of the ketogenic diet in modulating certain metabolic, neurological, and inflammatory disorders such as type II diabetes, epilepsy, osteoporosis, and cancer suggesting the potential therapeutic benefit (Feinman et al., 2015; Ferrere et al., 2021). However, this dietary therapeutic strategy, based on carbohydrate restriction or fasting for achieving high circulatory levels of BHB, had limited clinical applications because of the reported GIT adverse effects alongside the difficulty in adherence to such low-carb dietary modifications (Batch et al., 2020; Masood et al., 2023).

Therefore, BHB could be exogenously supplemented to the body for the speedy attainment of temporary ketosis without dietary restrictions. This notion is supported by earlier studies that have reported the good safety profile of exogenous BHB and have acknowledged its anti-inflammatory and antioxidative properties (Neudorf et al., 2020; Huang et al., 2022).

In addition to the potential therapeutic effects of BHB, previous discussions have highlighted the physiological functions of BHB and other ketone bodies, emphasizing their fundamental role in signaling transduction and their significance as essential energy substrates during periods of starvation. Moreover, Recent research has shed light on the beneficial role of BHB in preventing inflammation and inhibiting the development of cancer. This effect was ascribed to BHB-mediated activation of G-protein coupled receptor (GPR109a) with subsequent suppression of the NFκB signaling axis (Youm et al., 2015; Li et al., 2021).

NLR family pyrin domain containing 3 (NLRP3) inflammasome is a sensor of innate immunity, that is expressed as inactive monomers. NLRP3 activation requires recruitment and assembly of three domains, namely, sensor protein, apoptosis-associated speck-like protein containing a caspase recruitment domain (ASC) known as the adaptor molecule, followed by pro-caspase-1 (effector molecule) leading to NLP3 polymerization and activation of the inflammasome. Subsequently pro-caspase-1 self cleaves into caspase-1that in turn stimulates cytokines activation (IL-1β and IL-18) as well as Gasdermin D (GSDMD) cleavage into NGSDMD triggering NLRP3/GSDMD mediated-pyroptosis. Pyroptosis is a type of programmed cell death executed by inflammatory cytokines (He et al., 2015).

Peculiar NLRP3 activation has been implicated in various inflammatory disorders such as pulmonary fibrosis, gout, and Alzheimer’s disease (Zohny et al., 2022b; Abdelhady et al., 2023). Moreover, the aforementioned literature has emphasized the involvement of the NLRP3 inflammasome in the progression of UC. Moreover, studies conducted on mice with NLRP3 knockout have shown their resistance to colitis. Furthermore, a recent investigation has highlighted the significance of inhibiting TNFα/NLRP3/IL-1β/caspase-1 in alleviating UC, with their findings demonstrated both *in vivo* and *in vitro* (Ning et al., 2023).

This study represents the first comparative report aiming to evaluate the therapeutic potential of a ketogenic diet compared to exogenous BHB in alleviating UC in a dextran sodium sulfate (DSS)-induced colitis model. The study focused on assessing the inhibition of the NFκB/NLRP3 crosstalk and its consequences of apoptosis and pyroptosis as the underlying mechanism, as well as exploring the effects of both therapeutic strategies on autophagy and the composition of the gut microbiota.

## 2 Materials and methods

### 2.1 Animals

Seven-week-old male Sprague Dawley rats (180–200 g) were obtained and then kept under standard conditions (22°C ± 2°C, relative humidity of 50% ± 10%, and a 12-h light/dark cycle) to acclimatize for 2 weeks before starting the experiment. Animals were fed *ad-libitum* and allowed unlimited access to drinking water. The protocol was approved by the IACUC under FPDU24120,3. All animals were treated and sacrificed according to the corresponding guidelines.

### 2.2 Chronic colitis induction

Chronic colitis was induced via administration of 2% DSS [in autoclaved drinking water (w/v)] (Sigma-Aldrich, St. Louis, MO, United States; Mw = ∼40,000) for 1 week, followed by 1% DSS for 10 days. Eventually, 2% DSS was administered for another 7 days (Hoffmann et al., 2017; Saber et al., 2023). The body weight of experimental animals was recorded daily and the percentage of body weight loss was calculated with respect to their original body weight recorded on the first day of the experiment.

### 2.3 Experimental design

As shown in Table 1, experimental animals were randomly assigned into six groups. The Normal group (*n* = 6) served as the control group. The KD group (*n* = 6), rats that were fed a ketogenic diet. The BHB group (*n* = 6), rats that were administered BHB (300 mg/kg/day; i.p.; Sigma-Aldrich) while being maintained on standard rodent food *ad libitum*. The cColitis group (*n* = 15), rats that received DSS only. The cColitis/KD group (*n* = 12), rats received DSS and were fed the ketogenic diet protocol. The cColitis/BHB group (*n* = 12), rats received DSS and the BHB (300 mg/kg/day; i.p.) while being maintained on standard rodent food *ad libitum*. On day 25 of the experiment, rats were sacrificed under thiopental anesthesia (40 mg/kg). Moreover, blood samples were collected from each animal group mainly for the assessment of BHB plasma levels. Before the animals’ sacrifice, both disease activity and body weight changes were evaluated in all groups. Then animals were sacrificed and tissue was harvested.

**TABLE 1:** Experimental design.

Exp. Groups	Days 1–7	Days 8–17	Days 18–24	Day 25
Normal group (*n* = 6)	Standard food	Standard food	Standard food	Sacrifice day
KD (*n* = 6)	Ketogenic diet	Ketogenic diet	Ketogenic diet	
BHB (*n* = 6)	Standard food	Standard food	Standard food	
	BHB (300 mg/kg/day; i.p.)	BHB (300 mg/kg/day; i.p.)	BHB (300 mg/kg/day; i.p.)	
cColitis (*n* = 15)	Standard food	Standard food	Standard food	
	2% DSS	1% DSS	2% DSS	
cColitis/KD (*n* = 12)	Ketogenic diet	Ketogenic diet	Ketogenic diet	
	2% DSS	1% DSS	2% DSS	
cColitis/BHB (*n* = 12)	Standard food	Standard food	Standard food	
	2% DSS	1% DSS	2% DSS	
	BHB (300 mg/kg/day; i.p.)	BHB (300 mg/kg/day; i.p.)	BHB (300 mg/kg/day; i.p.)	

BHB, β-hydroxybutyrate; cColitis, chronic colitis; DSS, dextran sodium sulfate; KD, ketogenic diet.

### 2.4 Rational of BHB dosing and the KD protocol

The control group, BHB group, and colitis/BHB group of rats were given unrestricted access to standard rodent food (FPDU rodent food). On the other hand, the rats in the KD group received FPDU KD rodent food, which contained 5% of calories from carbohydrates, 80% from fat, and 15% from protein (FPDU approved KD; 6 kcal/g; Delta University for Science and Technology, Gamasa, Egypt). The fat sources in the KD consisted of a combination of soybean oil and lard, providing a mixture of saturated and unsaturated long-chain fatty acids.

Additionally, a preliminary study showed that the KD rodent food resulted in a significant increase in plasma levels of BHB (>1 mmol/L) compared to normal rats (*p* = 0.0001) after continuous feeding for 7 days. These elevated levels are typically indicative of nutritional ketosis in most individuals following a KD. To maintain BHB plasma levels above 1 mmol/L, exogenous BHB was administered intraperitoneally at a dose of 300 mg/kg every day at 8:00 a.m. until the last day of the experiment. After 8 h of its injection, BHB plasma levels remained higher than the threshold (*p* < 0.0001 compared to the normal group). Water consumption was carefully monitored to ensure consistent exposure to DSS across all rat groups, as it is crucial for obtaining accurate and reproducible results in this colitis model. We observed that a 200 g SD rat consumed 25–30 mL of drinking water/day and those receiving DSS consistently consumed 20–25 mL of 2% DSS solution/day. The quantity of food consumed is also monitored, and it was observed that a 200 g SD rat consistently consumed approximately 15 g of food per day across all experimental groups. However, alterations in water and food consumption occur in response to variations in the concentration of DSS and the duration of exposure, resulting in an aversion to both food and water.

### 2.5 Determination of the colonic weight/length ratio

The colonic weight/length ratio was assessed mainly for estimating the severity of UC. This approach was conducted by measuring the length of the entire colon. Then the ratio of the colon’s weight to its length was calculated. The obtained results were expressed in grams per centimeter of colonic tissue.

### 2.6 Determination of the disease activity index

The disease activity index (DAI) is a widely accepted method used in preclinical research to evaluate the severity of colitis by quantitatively assessing disease symptoms. On the final day of the study, a blinded gastroenterologist evaluated each rat and assigned an average DAI score based on the severity of specific symptoms, including the percentage decrease in body weight, presence of diarrhea, and observation of bloody feces. The DAI score was determined using predetermined criteria that are commonly employed in such assessments (Table 2) (Youssef et al., 2021). The maximum average score is 3.33.

**TABLE 2:** Evaluation criteria for the disease activity index (DAI).

Score	% decrease in body weight	Score	Stool consistency	Score	Detection of blood in the stool
0	No change	0	Normal consistency	0	Negative
1	1%–5%	1	Soft	1	Positive hemoccult
2	6%–10%	2	Very soft	2	Traces of blood
3	11%–15%	3	Watery diarrhea	3	Gross rectal bleeding
4	16%–20%				

### 2.7 Determination of the macroscopic damage index (MDI)

MDI score was assessed for each animal by a specialized blinded pathologist following examination of the macroscopic pathologic features of the spotted injuries in colon segments opened longitudinally (Youssef et al., 2021). The MDI scoring criteria are summarized in Table 3. The maximum score is 10.

**TABLE 3:** Scoring criteria for the macroscopic damage index (MDI).

Score	Macroscopic features
1	No damage
2	Hyperemia but no ulcers detected
3	Linear ulcer detection/No remarkable inflammation
4	Two or more sites with inflammation or ulceration
5	Two or more spots of inflammation or ulceration or one spot of inflammation or ulceration covering a distance longer than 1 cm length of the colon
6	Inflammation or ulceration spot that covers 2 cm length of the colon. Additionally, each 1 cm increase in the length of the colonic lesion equates increase in the score by one

### 2.8 Colon tissue sample collection and DNA extraction

The colons of the rats were dissected and weighed, and their lengths were measured. Additionally, plasma was isolated to determine the level of BHB. The colons were then rinsed with ice-cold solution and dried using sterile pads. They were divided into two parts: the first part was immediately frozen in liquid nitrogen and stored at −80°C for further biochemical analysis after homogenization. The second part, which included the distal colon, was placed in neutral-buffered formalin (10%) for histopathological examination (Thavarajah et al., 2012). Stool samples were collected from the cecum (300 mg) of each rat immediately after the dissection step. DNA was extracted from the fecal specimens using the QIAamp DNA Stool Mini Kit (Qiagen Inc., Germany) following the manufacturer’s guidelines. The concentration of DNA was determined using a NanoDrop (OPTIZEN NanoQ, Mecasys).

### 2.9 Histological examination

The colon tissues were thoroughly rinsed using distilled water followed by extensive dehydration using serial dilutions of ethanol. Then, tissue samples were washed with xylene and fixed in paraffin at 56°C for preparing paraffin tissue blocks that were then cut into sections (4–5-μm thick) using a microtome (Youssef et al., 2022). Eventually, specimens were processed and stained with hematoxylin & eosin stain and the specimens were examined using a Leica DFC camera. The standard histology procedures were conducted blindly by a histologist. Then, histological scoring was determined for each tissue sample as illustrated in Table 4. The maximum score is 6.

**TABLE 4:** Histological scoring criteria.

Score	Microscopic features				
0	Normal histopathologic findings				
1	Mucosal inflammation and/or focal ulceration				
2	Focal or extensive mucosal and submucosal inflammation and/or ulceration				
3	Focal or extensive inflammation and/or ulceration extends to the muscularis propria				
4	Focal ulceration and transmural inflammation extend to the serosa				
5	Extensive ulceration and transmural inflammation extend to the serosa				
6	Focal or extensive ulceration and transmural inflammation and perforation				

### 2.10 Determination of ROS, malondialdehyde (MDA), superoxide dismutase (SOD), and reduced glutathione (GSH)

For the determination of ROS levels in colon tissues, 200 mg of fresh colon samples were homogenized in ice-cold Tris-HCl buffer (40 mM, pH = 7.4) at a 1:10 w/v ratio. Afterward, 100 μL of obtained tissue homogenate was mixed with 1 mL of Tris-HCl buffer followed by adding 10 μM of 2′,7′-dichlorofluorescin diacetate (Sigma). The mixture was incubated at 37°C for 30 min, then fluorescence intensity (FI) was measured (excitation at 485 nm and emission at 525 nm) using a SpectraFluor Plus Microplate Reader (Tecan, Mainz, Germany).

Assessment of MDA level in colon tissue homogenate was carried out using a quantitative analysis kit obtained from Bio-diagnostic (Giza, Egypt), following the manufacturer’s instructions. The reaction involved incubating the homogenate with thiobarbituric acid (95°C for 30 min). This reaction yielded a thiobarbituric acid reactive product, which was measured colorimetrically (534 nm).

As per SOD and GSH, their levels in colon tissue homogenate were assayed using the corresponding quantitative analysis kits (Bio-diagnostic, Egypt), and the manufacturer’s instructions were carefully followed.

GSH assay involved the reduction of 5,5′ dithiobis (2–nitrobenzoic acid) (DTNB) using GSH to yield a yellow reduced chromogen whose absorbance (Ab) was measured at 405 nm. The concentration of GSH was calculated in terms of the recorded Ab since it was directly proportional to GSH concentration. All assays of oxidative stress markers were determined in duplicate.

### 2.11 Determination of plasma BHB

Plasma samples were centrifuged (10,000 g for 10 min), then the supernatant was aspirated and subjected to a second round of centrifuging the supernatants at the same speed. The second supernatant was ultrafiltered (50 KD ultrafiltration tube for 15 min). In this assay (Elabscience, Wuhan, China), the BHB dehydrogenase enzyme catalyzes BHB oxidation, associated with NAD^+^ reduction to NADH which transfers electrons to WST-8 yielding a yellow product. The content of BHB was calculated by measuring the absorbance value at 450 nm. The BHB assay was determined in duplicate.

### 2.12 Determination of tumor necrosis factor-alpha (TNF-α), IL-6, IL-10, IL-1β, and IL-18 levels in colon tissue

ELISA kits were used for assessing the levels of the following inflammatory parameters: IL-10 and TNF-α (LifeSpanBioSciences, Inc., Seattle, WA, United States), IL-6 (R&D System, Minneapolis, MN, United States), IL-1β (BioLegend, San Diego, CA, United States), IL-18 (eBioscience, Vienna, Austria), following manufacturers’ instructions. All cytokine assays were determined in duplicate.

### 2.13 Determination of myeloperoxidase (MPO) activity, NFκB DNA binding activity, caspase-1 activity, and active caspase-3

Myeloperoxidase is a leukocyte-derived peroxidase enzyme that is central to tissue inflammation. The activity of MPO in rats’ colons was assessed using an MPO assay (Sigma-Aldrich) kit. The amount of MPO enzyme that can hydrolyze the substrate to produce taurine chloramine and consume 1.0 mol of TNB per minute at 25°C is defined as one unit of MPO activity. The MPO assay was done in duplicate.

Nuclear translocation of the p65 subunit was conducted using the respective assay kit (Abcam, United States). The kit operated a specific double-stranded DNA sequence containing the NFκB p65 consensus binding site (5′–GGGACTTTCC–3′) to bind to the active NFκB p65. Upon binding of the active protein to the target DNA, the epitope of NFκB p65 becomes accessible and was detected by a primary antibody. The NFκB p65 activity was determined in duplicate.

A caspase-1 colorimetric assay kit from R&D Systems was used for assaying caspase-1 activity. A microtiter plate reader was used to measure the p-nitroanilide (p-NA) light emission (405 nm) after the chromophore was separated from the labeled substrate YVAD-p-NA. Tissue lysates were prepared using cold lysis buffer and then centrifuged (10,000 × g) releasing cytosolic extracts. Then the protein concentration was quantified. Later 50 μL of lysis buffer was mixed with 100 μg of protein. Then YVAD-p-NA substrate (5 μL) and the reaction buffer (50 µL) were added to each sample followed by 1–2 h incubation (at 37°C). Finally, the samples were assayed in duplicate and read at 405 nm in a microtiter plate reader.

The active caspase-3 levels were measured using a kit obtained from MyBioSource Inc. (San Diego, CA, United States). The kit utilized a polyclonal anti-active caspase-3 antibody and an active caspase-3-HRP conjugate. The color intensity obtained is inversely proportional to the amount of active caspase-3 present in the samples. The competition between the active caspase-3 from the samples and the active caspase-3-HRP conjugate for binding sites on the anti-active caspase-3 antibody was limited due to the limited number of available binding sites. Therefore, as more binding sites were occupied by active caspase-3 from the sample, fewer binding sites remained available for active caspase-3-HRP conjugate to bind. The determination of active caspase-3 was conducted in duplicate.

### 2.14 RT-qPCR analysis for the mRNA expression of ASC and NLRP3

Total RNA was extracted from colonic tissues using a Qiagen kit (Venlo, the Netherlands), following the supplier’s instructions. NanoDrop (Thermo Fisher Scientific, United States) was used for the evaluation of RNA quality and purity (260 nm). Reverse RNA transcription was conducted using the RevertAid First Strand cDNA synthesis kit. Moreover, StepOne™ Real-Time PCR System (Thermo Fisher Scientific) was used for performing RT-qPCR. The comparative cycle threshold (Ct) (2^−ΔΔCT^) method was used for calculating relative gene expression levels of ASC and NLRP3 normalized to the GAPDH gene. The PCR primer pairs used are described in Table 5 (He et al., 2021; He et al., 2023).

**TABLE 5:** Sequence of ASC, NLRP3 and GAPDH primer pairs.

Gene	Primer sequence (5′-3′)
ASC	F: 5′- CTC​TGT​ATG​GCA​ATG​TGC​TGA​C-3′
	R: 5′- GAA​CAA​GTT​CTT​GCA​GGT​CAG-3′
NLRP3	F: 5′- GAG​CTG​GAC​CTC​AGT​GAC​AAT​GC-3′
	R: 5′- ACC​AAT​GCG​AGA​TCC​TGA​CAA​CAC-3′
GAPDH	F: 5′- TCA​AGA​AGG​TGG​TGA​AGC​AG-3′
	R: 5′- AGG​TGG​AAG​AAT​GGG​AGT​TG-3

### 2.15 Determination of NLRP3, NGSDMD, BECN1, p62, ZO-1, OCLN, and CLDN5

Protein levels of NLRP3 and NGSDMD in colon tissue homogenates were estimated using the respective ELISA kit (MyBioSource Inc., San Diego, CA, United States). BECN1 and p62 colon tissue levels were determined using ELISA kits supplied by CUSABIO (Wuhan, China) and MyBioSource, respectively. All protocols followed the manufacturer’s instructions. ZO-1, OCLN, and CLDN5 levels were determined following instructions given by CUSABIO.

### 2.16 Detection of gut microbiota using conventional PCR and the determination of the relative abundance using RT-qPCR

Firstly, DNA preparation for thermal cycling involved mixing the following: 12.5 µL of my Taq red mix (Bioline Co., United Kingdom), 1 μL of each primer (10 µM each), 2.5 µL of DNA, and nuclease-free water. Later PCR amplification was achieved through preliminary denaturation (94°C for 5 min), then 35 cycles (94°C for 30 s), annealing at a temperature calculated for each primer mix for 30 s, extension at 72°C for 45 s, and a last termination step at 72°C for 3 min. Eventually, electrophoretic separation was conducted on a 1.5% agarose gel to detect the expected PCR amplicons and compared them with a GeneRuler 100 bp plus DNA ladder (Thermo Scientific, United States). The gels were stained with ethidium bromide and visualized using a UV transilluminator. Table 6 lists the specific primer sequences for each type of bacteria.

**TABLE 6:** Primer sequences for the detection of different species of bacteria.

Primer name		Primer sequence	Ta (°C)	bp
(16S)	F	GAG​TTT​GAT​CCT​GGC​TCA​G	51	312
	R	GCTGCCTCCCGTAGGAGT		
*Fusobacterium*	F	GGA​TTT​ATT​GGG​CGT​AAA​GC	51.5	162
	R	GGC​ATT​CCT​ACA​AAT​ATC​TAC​GAA		
*Bacteroides* spp.	F	AAG​GGA​GCG​TAG​ATG​GAT​GTT​TA	55	193
	R	CGA​GCC​TCA​ATG​TCA​GTT​GC		
*Clostridium* spp.	F	CGG​TAC​CTG​ACT​AAG​AAG​C	50	429
	R	AGT​TTG​ATT​CTT​GCG​AAC​G		
*Bifidobacterium*	F	CTCCTGGAAACGGGTGG	51	551
	R	GGT​GTT​CTT​CCC​GAT​ATC​TAC​A		
*Lactobacillus* spp.	F	AGC​AGT​AGG​GAA​TCT​TCC​A	50	334
	R	CACCGCTACACATGGAG		

To evaluate the relative abundance of specific bacteria in the gut, we employed primers for the 16S rDNA housekeeping gene. Fecal DNA (40–80 ng) was extracted and combined with 12.5 μL (2x) SYBR Green PCR master mix (Willowfort Co., Birmingham, United Kingdom), 1.5 μL of each forward and reverse primer (10 μmol), and 7.5 μL of nuclease-free water, resulting in a final volume of 25 μL. Real-time PCR was performed on a MyGo machine using the following cycling protocol: an initial 5-min denaturation step at 95°C, followed by 45 cycles of 95°C for 20 s, annealing for 20 s, and 72°C for 40 s. The Ct values and melting curves were obtained using MyGo software. The relative abundance of each bacterial species was calculated as a relative unit normalized to the total bacteria in the corresponding sample, using the 2^−ΔΔCT^ method (where ΔCt represents the average Ct value of each target minus the average Ct value of total bacteria). The primer sequences for detecting the various bacterial strains are listed in Table 6 (Saber et al., 2021a).

### 2.17 Statistical analysis

The statistical analysis was carried out using GraphPad Prism software version 9 (GraphPad Software Inc., La Jolla, CA, United States). The results were expressed as mean ± standard deviation (SD). One-way analysis of variance (ANOVA) was performed followed by Tukey’s *post hoc* test to determine the differences between groups. All statistical tests were performed at a significance level of less than 0.05. Pairwise comparisons provide information about the significance levels between different groups. In this context, symbols are used to indicate the level of statistical significance. Typically, one symbol (*) denotes a *p*-value less than 0.05, two symbols (**) denote a *p*-value less than 0.01, three symbols (***) denote a *p*-value less than 0.001, and four symbols (****) denote a *p*-value less than 0.0001.

## 3 Results

### 3.1 Effects of exogenous BHB and KD on the microscopic features of DSS-induced colitis in rats

**FIGURE 1:**
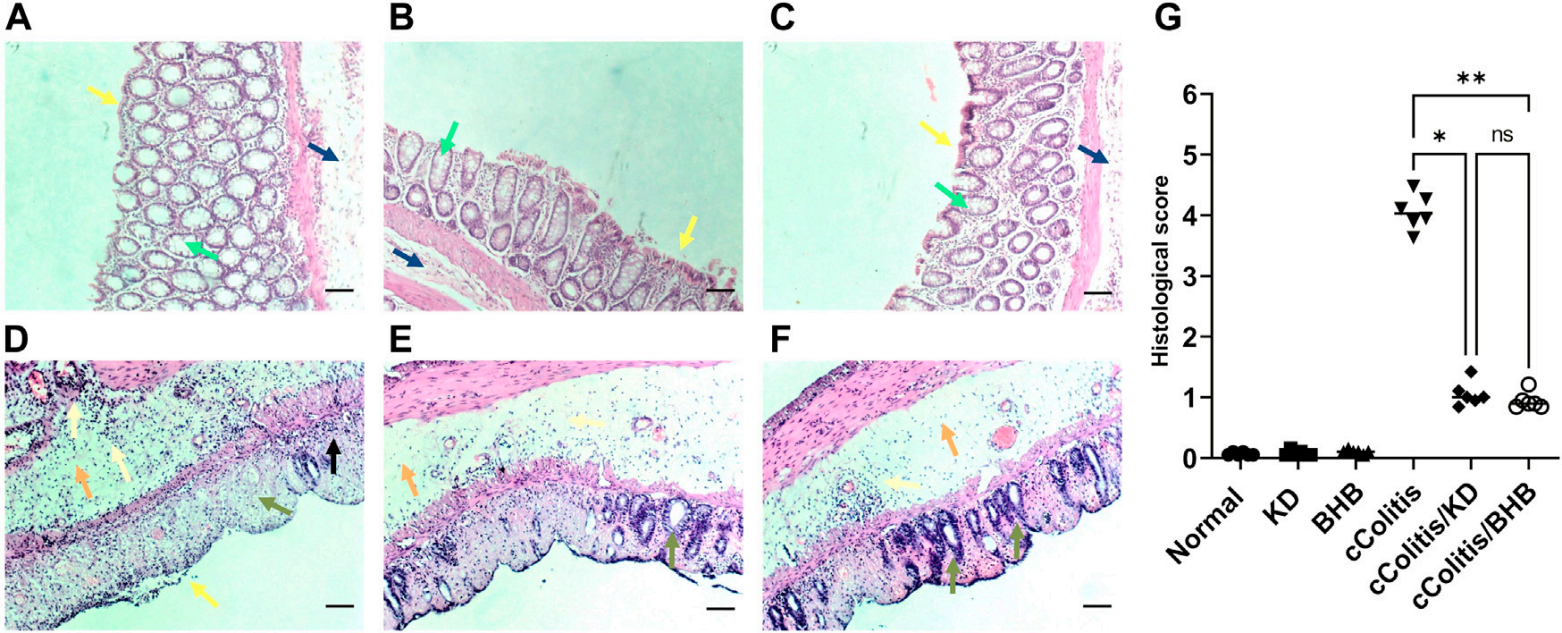
The figure illustrates the results of histopathological assessments performed on four groups: Normal **(A)**, KD **(B)**, BHB **(C)**, and cColitis **(D)**, as well as two subgroups within the cColitis group: cColitis/KD **(E)** and cColitis/BHB **(F)**. The Normal, KD, and BHB groups displayed normal epithelial layer (yellow arrow), and mucosal architecture with well-arranged and regularly shaped mucus-secreting colonic glands containing goblet cells (green arrow) as well as a normal submucosal layer (blue). In contrast, the cColitis group showed signs of inflammation, including erosions and de-epithelialization (yellow arrow), submucosal edema (orange arrow), distorted architecture, destruction of colonic glands, and loss of goblet cells (green arrow). Inflammatory infiltrates of various types of immune cells were also observed in the mucosal (black arrow) and submucosal regions which are also extended to muscularis propria (gold arrows). The cColitis/KD and cColitis/BHB subgroups showed improvements in mucosal structure, with the appearance of glands and crypts (green arrows), reduced inflammatory cell infiltrate (gold arrows), and a clear submucosa with low levels of inflammation (orange arrows). Although edema was slightly improved, there was less submucosal congestion and collagen deposition in these subgroups. The inflammatory score assessment **(G)** indicated that exogenous BHB was more effective than KD in reducing inflammation induced by DSS in the colon. Scale bar = 100 µm. (Control and BHB groups: *n* = 6, cColitis group: *n* = 15, cColitis/BHB & cColitis/KD group: *n* = 12). Statistical analysis was performed by one-way ANOVA followed by Tukey’s *post hoc* test. *p < 0.05, **p < 0.01, ns = no significance.

Figure 1 illustrates the results of histopathological assessments performed on four groups: Normal (A), KD (B), BHB (C), and cColitis (D), as well as two subgroups within the cColitis group: cColitis/KD (E) and cColitis/BHB (F). The Normal, KD, and BHB groups displayed normal mucosal architecture with well-arranged and regularly shaped mucus-secreting colonic glands containing goblet cells. In contrast, the cColitis group showed signs of inflammation, including submucosal edema, distorted architecture, destruction of colonic glands, and loss of goblet cells. Inflammatory infiltrates of various types of immune cells were also observed in the mucosal and submucosal regions. The cColitis/KD and cColitis/BHB subgroups showed improvements in mucosal structure, with the appearance of glands and crypts, reduced inflammatory cell infiltrate, and a clear submucosa with low levels of inflammation. Although edema was slightly improved, there was less submucosal congestion and collagen deposition in these subgroups. Furthermore, inflammatory score assessment (G) revealed that exogenous BHB was more effective than KD in reducing the histological score.

### 3.2 Effects of exogenous BHB and KD on the % weight change, and the colon wt/length ratio

**FIGURE 2:**
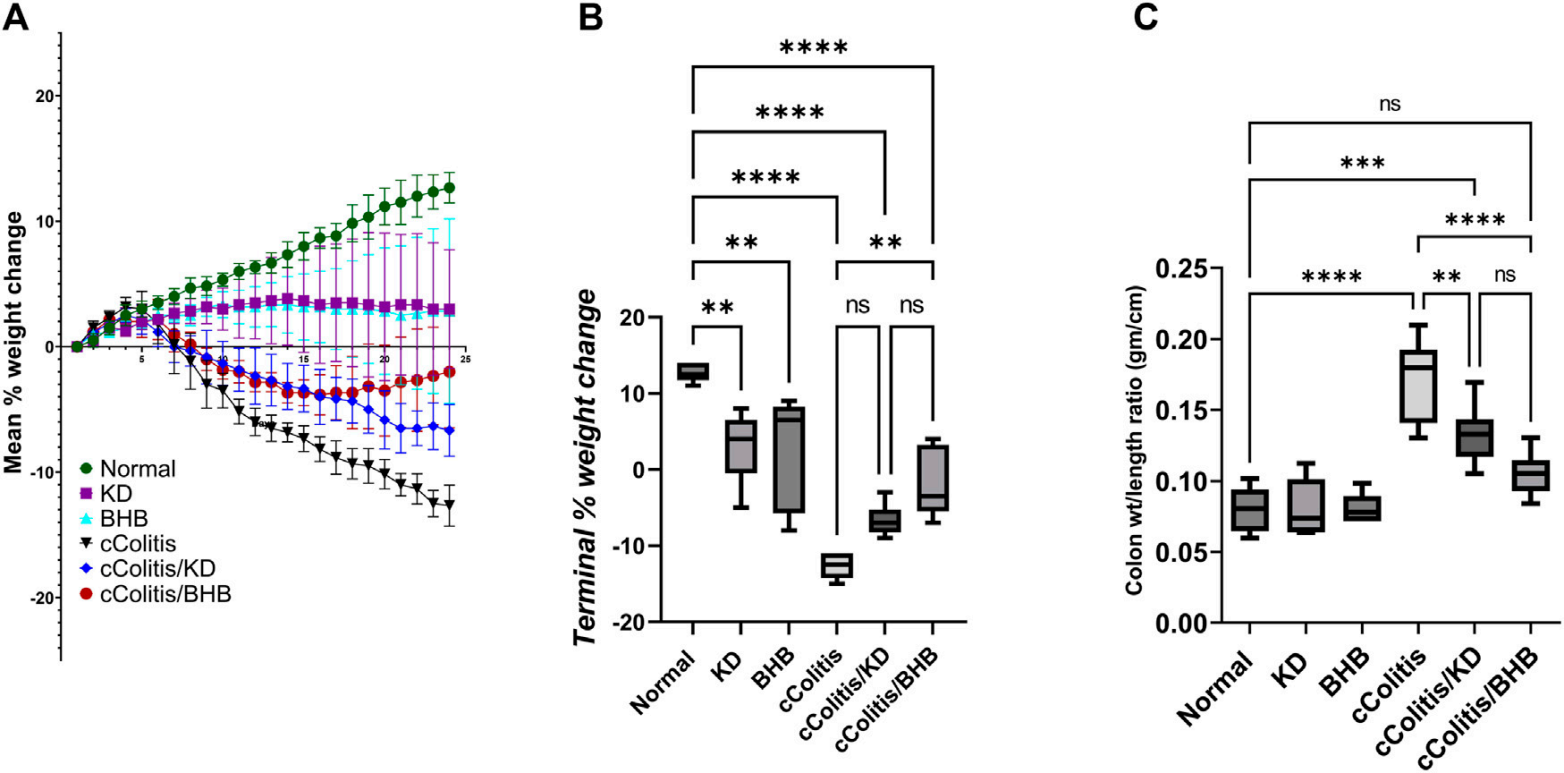
Effects of exogenous BHB and KD on the mean % weight change **(A)**, terminal % weight change **(B)**, and the colon wt/length ratio **(C)** in DSS-induced chronic colitis in rats. These findings suggest that BHB supplementation attenuated weight loss in the colitis-induced group, while both KD and BHB interventions impacted the colon weight-to-length ratio in the experimental model. Data are presented as mean ± SD. Significance between groups is indicated by pairwise comparisons. (Control and BHB groups: *n* = 6, cColitis group: *n* = 15, cColitis/BHB & cColitis/KD group: *n* = 12). Statistical analysis was performed by one-way ANOVA followed by Tukey’s *post hoc* test. **p < 0.01, ***p < 0.005, ****p < 0.001, ns = no significance.

The daily assessment of body weight was conducted on rats in all experimental groups. The average percentage weight change (Figure 2A) and the final percentage weight change (Figure 2B) were calculated. The percentage of weight loss in the cColitis group was found to be significantly greater than that in the normal animals. In Figure 2B, it is evident that the cColitis/BHB group exhibited a significantly lower percentage of weight loss compared to the cColitis group. However, the cColitis/KD group did not show a significant difference in percentage weight loss when compared to the cColitis group. This lack of significance may be attributed to the weight loss potential and the counteractive effect of KD on weight gain. Regarding the impact of the experimental interventions on the colon weight-to-length ratio (gm/cm) (Figure 2C), it is noteworthy that both KD and BHB administration resulted in a significant change in the colon weight-to-length ratio compared to the cColitis group. Additionally, the cColitis group exhibited a significantly higher colon weight-to-length ratio compared to the normal animals.

### 3.3 Effects of exogenous BHB and KD on disease activity index and macroscopic damage index

**FIGURE 3:**
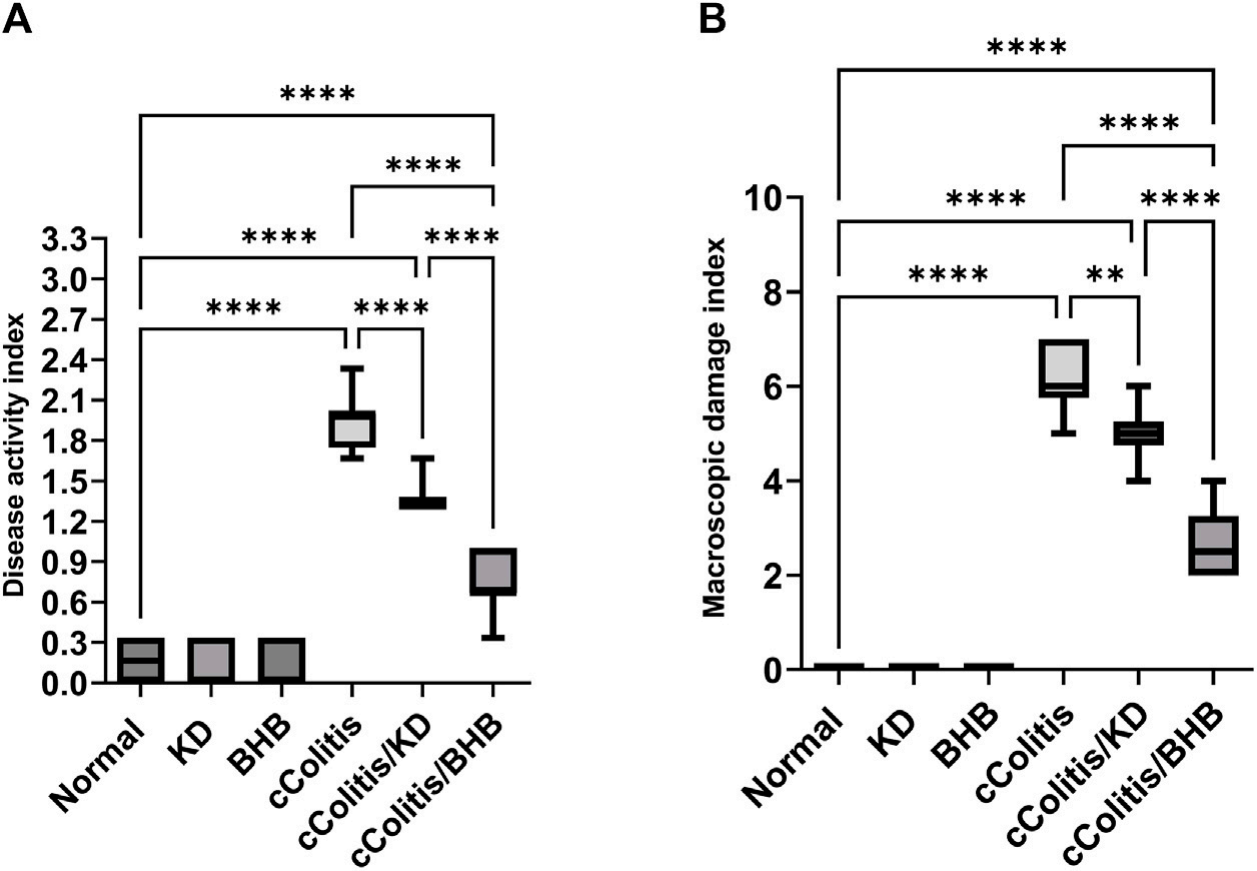
Effects of exogenous BHB and KD on disease activity index **(A)** and macroscopic damage index **(B)**. These findings suggest that both KD and BHB interventions have a protective effect against colonic inflammation induced by DSS, with BHB showing a more pronounced effect in reducing disease activity and macroscopic damage indices. Significance between groups is indicated by pairwise comparisons. (Control and BHB groups: *n* = 6, cColitis group: *n* = 15, cColitis/BHB & cColitis/KD group: *n* = 12). Statistical analysis was performed by one-way ANOVA followed by Tukey’s *post hoc* test. **p < 0.01, ****p < 0.001.

First and foremost, the rats in the normal control group did not exhibit any signs of colonic damage, such as inflammation or necrosis. The results of the present study also demonstrate that feeding the animals with KD or BHB did not induce any significant changes in disease activity or macroscopic damage indices, as depicted in Figures 3A, B, respectively. However, the administration of DSS resulted in a significant increase in both disease activity and macroscopic damage indices compared to the control rats. Moreover, treating the animals with KD or BHB after DSS exposure remarkably suppressed both disease activity and macroscopic damage indices. Notably, the DAI and the MDI values in the cColitis/BHB group were significantly reduced compared to those of the cColitis/KD group, indicating a greater benefit in alleviating colonic inflammation.

### 3.4 Effects of exogenous BHB and KD on colonic oxidative stress biomarkers (ROS, MDA, SOD, GSH)

**FIGURE 4:**
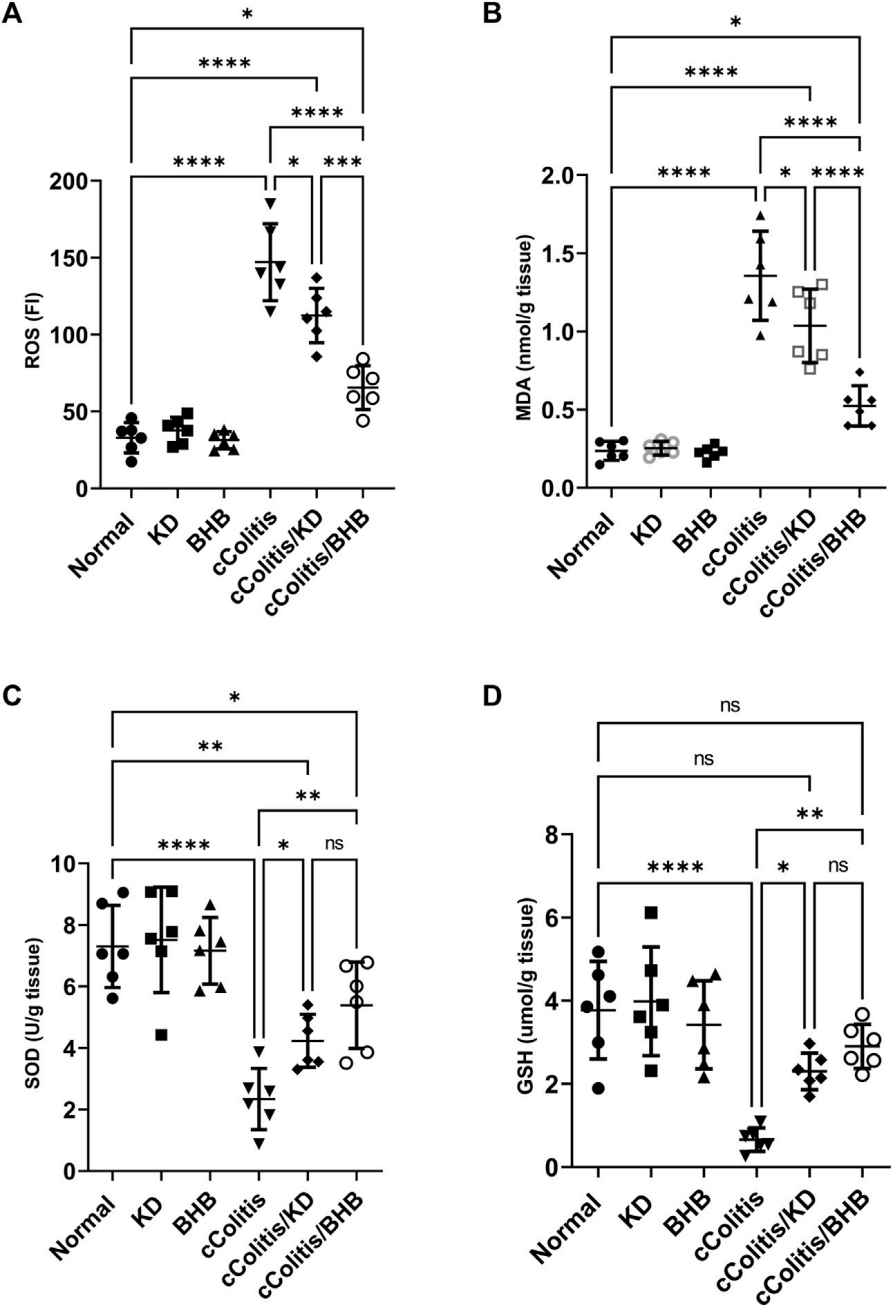
Effects of exogenous BHB and KD on colonic oxidative stress biomarkers ROS **(A)**, MDA **(B)**, SOD **(C)**, and GSH **(D)**. These findings indicate that both KD and BHB interventions effectively attenuated oxidative stress induced by DSS treatment. Although KD and BHB did not exert significant effects on the basal levels of oxidative stress biomarkers, they demonstrated potent antioxidant properties when the colon was exposed to DSS-induced oxidative stress. Data are presented as mean ± SD. Significance between groups is indicated by pairwise comparisons. (Control and BHB groups: *n* = 6, cColitis group: *n* = 15, cColitis/BHB & cColitis/KD group: *n* = 12). Statistical analysis was performed by one-way ANOVA followed by Tukey’s *post hoc* test. *p < 0.05, **p < 0.01, ***p < 0.005, ****p < 0.001, ns = no significance.

As depicted in Figure 4, the data from this study revealed that DSS treatment induced oxidative stress in the colon, as evidenced by a significant increase in colonic ROS (A) and MDA (B) content. Additionally, there was a noticeable suppression in the levels of antioxidants, including SOD (C) and GSH (D), compared to the control rats. In contrast, neither KD feeding nor BHB administration caused any significant changes in the aforementioned biomarkers. However, both the cColitis/KD group and cColitis/BHB group demonstrated effective improvement in DSS-induced oxidative stress. This improvement was reflected by a significant reduction in colonic ROS and MDA production, along with a remarkable increase in SOD and GSH levels.

### 3.5 Effects of exogenous BHB and KD on BHB serum level

**FIGURE 5:**
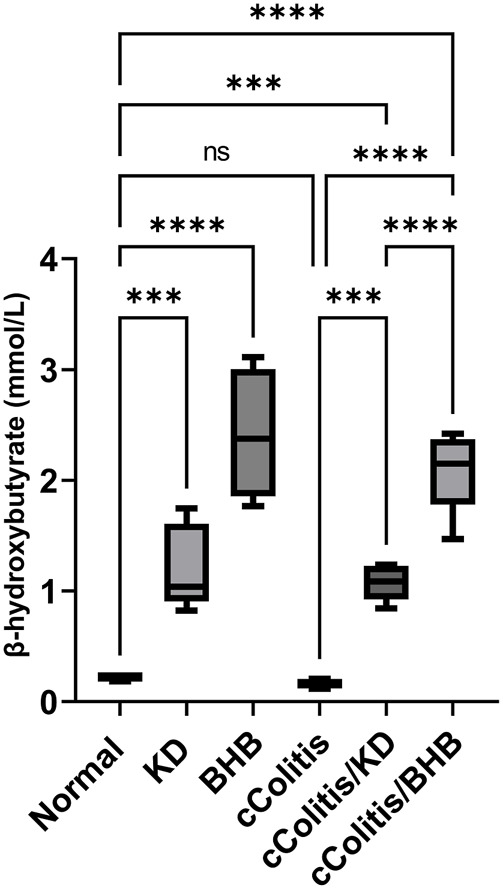
Effects of exogenous BHB and KD on BHB blood levels. Both KD feeding and BHB administration resulted in elevated plasma BHB levels ranging from 1–3 mmol/L, indicating a state of nutritional ketosis. Data are presented as mean ± SD. Significance between groups is indicated by pairwise comparisons. (Control and BHB groups: *n* = 6, cColitis group: *n* = 15, cColitis/BHB & cColitis/KD group: *n* = 12). Statistical analysis was performed by one-way ANOVA followed by Tukey’s *post hoc* test. ***p < 0.005, ****p < 0.001, ns = no significance.

Both the control animals and the cColitis group exhibited baseline plasma levels of BHB, as shown in Figure 5. Remarkably, both the KD and BHB administration resulted in elevated plasma BHB levels ranging from 1–3 mmol/L, indicating a state of nutritional ketosis. Moreover, rats that were fed with KD after DSS exposure displayed a significant increase in plasma BHB levels compared to the cColitis group. Importantly, the cColitis/BHB group demonstrated a remarkable elevation in plasma BHB levels, which was found to be statistically significant when compared to the cColitis/KD group. This significant change suggests that the coloprotective role of BHB is dose-dependent and may be correlated with the levels of BHB in the bloodstream. It also highlights the superiority of BHB as an injectable form over KD in this context.

### 3.6 Effects of exogenous BHB and KD on the level of inflammatory mediators (TNF-α, IL-6, IL10, IL-1β, and IL-18)

**FIGURE 6:**
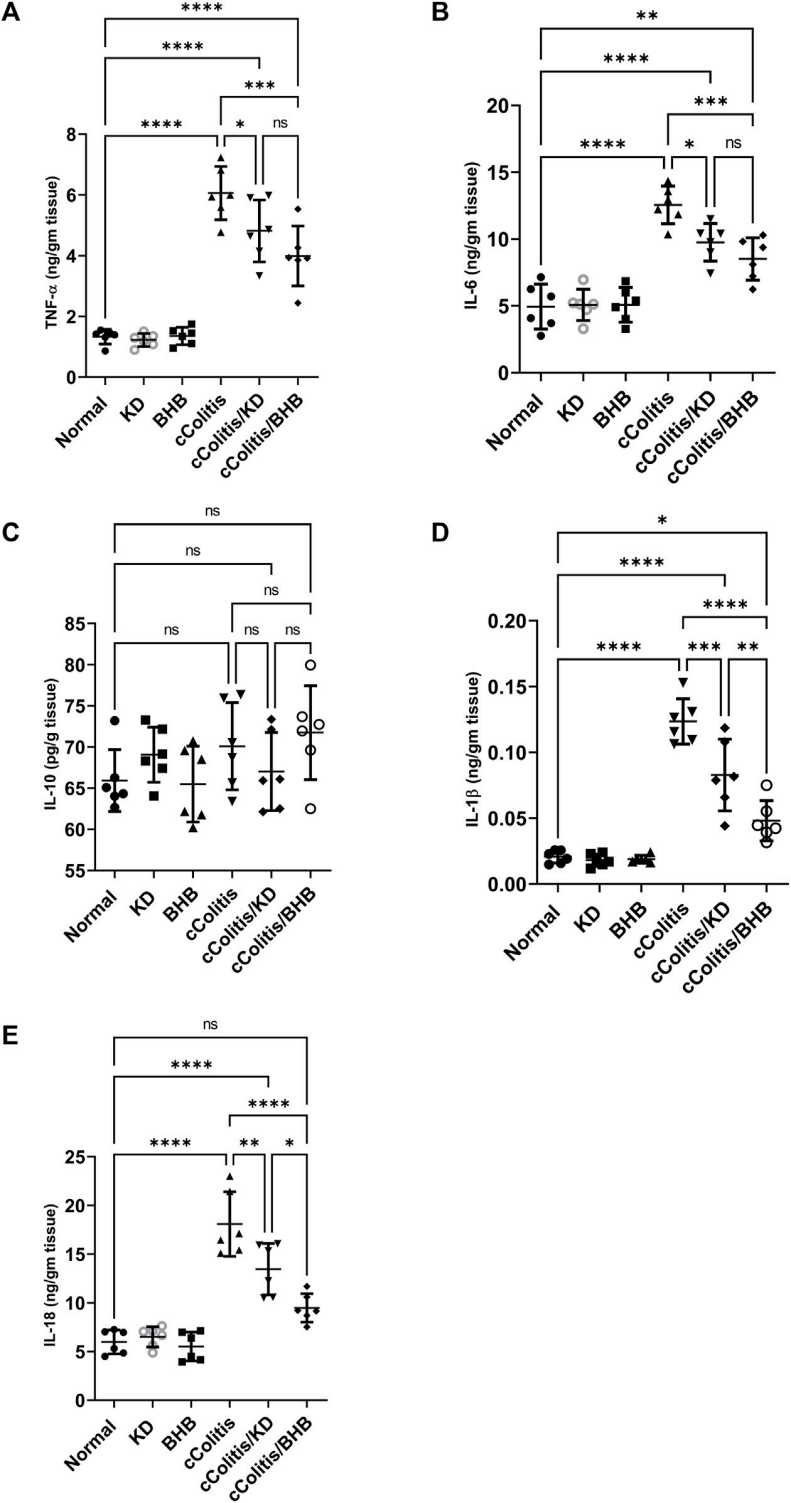
Effects of exogenous BHB and KD on the level of inflammatory mediators TNF-α **(A)**, IL-6 **(B)**, IL-10 **(C)**, IL-1β **(D)**, and IL-18 **(E)**. These findings highlight the potential of both KD and BHB in modulating colonic inflammation, as demonstrated by their impact on proinflammatory cytokines. The stable levels of IL-10 suggest the chronicity of the colitis model. Data are presented as mean ± SD. Significance between groups is indicated by pairwise comparisons. (Control and BHB groups: *n* = 6, cColitis group: *n* = 15, cColitis/BHB & cColitis/KD group: *n* = 12). Statistical analysis was performed by one-way ANOVA followed by Tukey’s *post hoc* test. *p < 0.05, **p < 0.01, ***p < 0.005, ****p < 0.001, ns = no significance.

As depicted in Figure 6, the administration of DSS resulted in notable inflammation in the colon, as indicated by a significant increase in the levels of the various proinflammatory cytokines TNF-α (A), IL-6 (B), IL-1β (D), and IL-18 (E) compared to the normal group. In contrast, both ketogenic diet (KD) feeding and administration of BHB effectively modulated the colonic inflammation induced by DSS, as evidenced by a significant decrease in the levels of inflammatory markers TNF-α, IL-6, IL-1β, and IL-18 compared to the diseased group. Interestingly, there were no significant differences observed among the groups, including the diseased group, in the levels of the anti-inflammatory cytokine IL-10 (C) compared to the normal group. This finding confirms the chronic nature of our model, as in chronic colitis, it is common for IL-10 levels to remain unchanged or even increased as a part of the body’s regulatory response to attenuate excessive immune response and limit inflammation.

### 3.7 Effects of exogenous BHB and KD on MPO activity, NFκB DNA binding activity, caspase-1 activity, and active caspase-3

**FIGURE 7:**
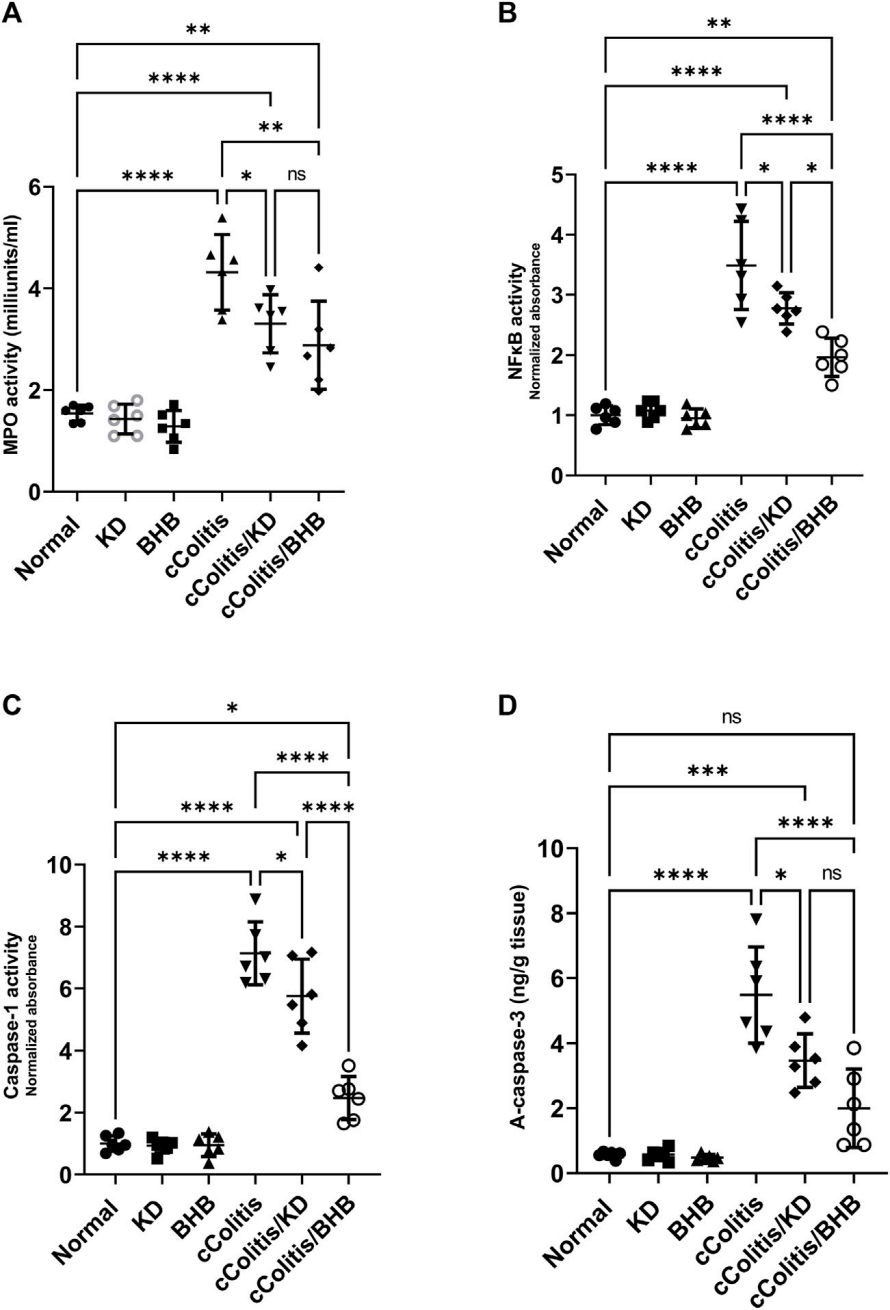
Effects of exogenous BHB and KD on MPO activity **(A)**, NFκB activity **(B)**, caspase-1 activity **(C)**, and active caspase-3 **(D)**. These findings suggest that both KD and BHB interventions have a protective effect against DSS-induced inflammation, as demonstrated by their ability to mitigate the alterations in inflammation-related parameters. BHB treatment showed superior efficacy in restoring NFκB binding activity and caspase-1 activity. Data are presented as mean ± SD. Significance between groups is indicated by pairwise comparisons. (Control and BHB groups: *n* = 6, cColitis group: *n* = 15, cColitis/BHB & cColitis/KD group: *n* = 12). Statistical analysis was performed by one-way ANOVA followed by Tukey’s *post hoc* test. *p < 0.05, **p < 0.01, ***p < 0.005, ****p < 0.001, ns = no significance.

Our study revealed that feeding rats a KD or exposure to BHB did not result in any significant alterations in several inflammation-related parameters, MPO activity, NFκB binding activity, caspase-1 activity, and active caspase-3 levels, when compared to the normal group. These findings are illustrated in Figures 7A–D, respectively. Conversely, the administration of DSS treatment led to a significant increase in the levels of these parameters compared to rats in the normal group. However, the detrimental effects induced by DSS exposure were substantially mitigated by both KD feeding and BHB treatment. Notably, BHB treatment exhibited greater efficacy than KD in restoring the aberrations caused by DSS treatment, particularly in NFκB binding activity and caspase-1 activity.

### 3.8 Effects of exogenous BHB and KD on the mRNA expression levels of NLRP3 and ASC and the protein levels of NLRP3 and NGSDMD

**FIGURE 8:**
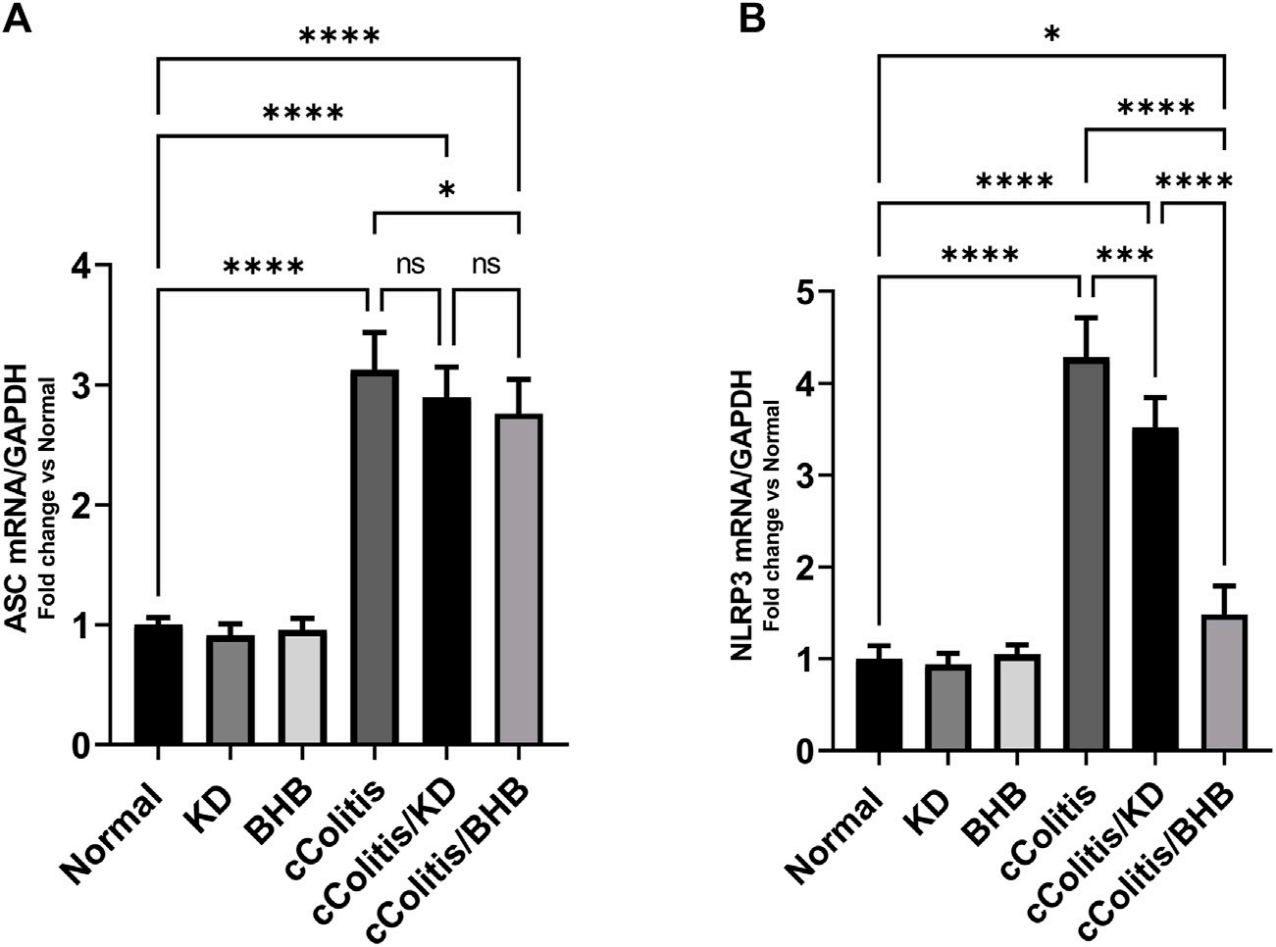
Effects of exogenous BHB and KD on the mRNA expression levels of ASC **(A)** and NLRP3 **(B)**. These findings suggest that both KD and BHB interventions have a modulating effect on specific parameters related to colonic inflammation induced by DSS treatment. BHB treatment demonstrated superior efficacy in reducing the levels of NLRP3 mRNA. Data are presented as mean ± SD. Significance between groups is indicated by pairwise comparisons. (Control and BHB groups: *n* = 6, cColitis group: *n* = 15, cColitis/BHB & cColitis/KD group: *n* = 12). Statistical analysis was performed by one-way ANOVA followed by Tukey’s *post hoc* test. *p < 0.05, ***p < 0.005, ****p < 0.001, ns = no significance.

**FIGURE 9:**
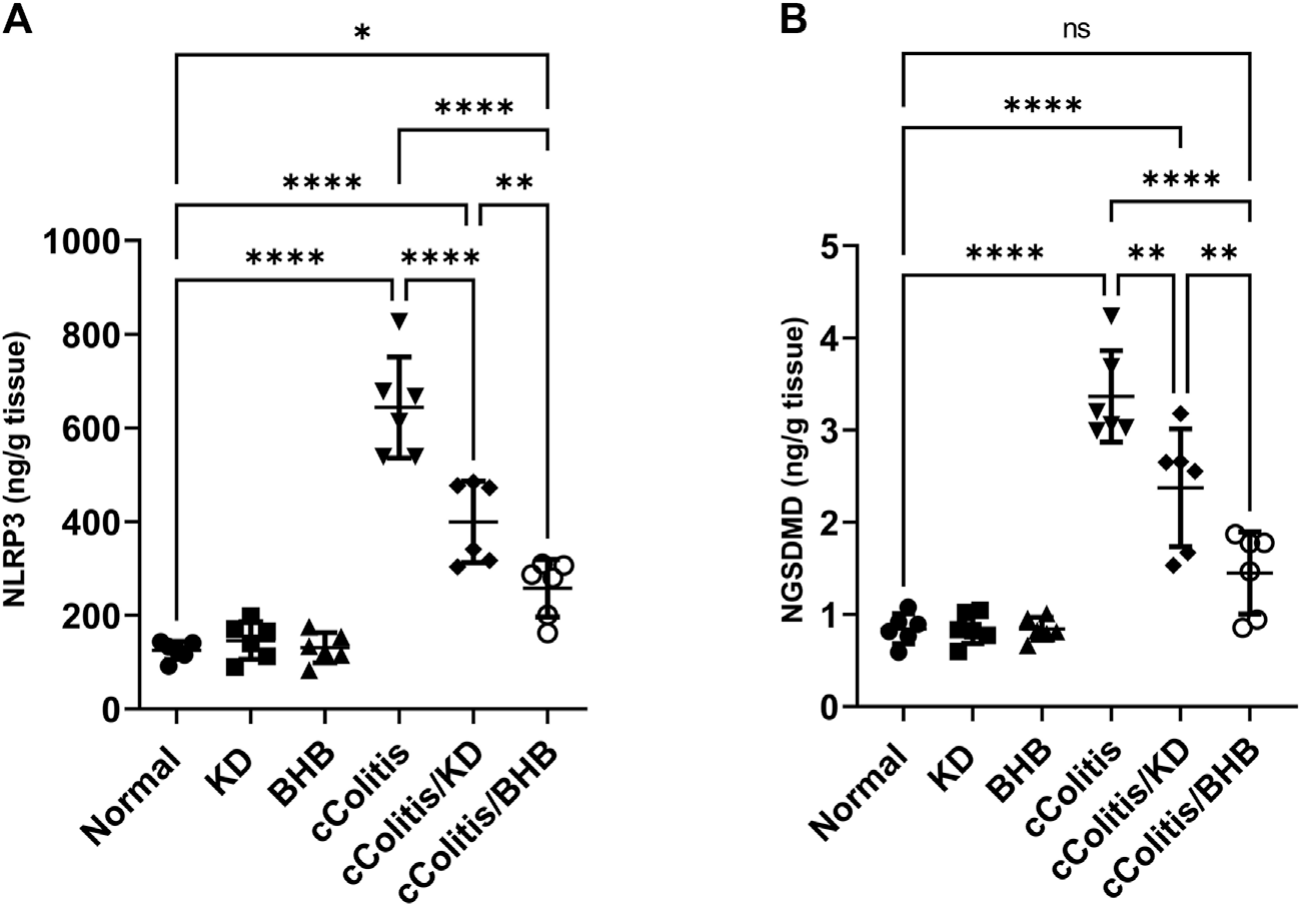
Effects of exogenous BHB and KD on the protein levels of NLRP3 **(A)** and NGSDMD **(B)**. These findings suggest that both KD and BHB interventions have a modulating effect on specific parameters related to colonic inflammation induced by DSS treatment. BHB treatment demonstrated superior efficacy in reducing the levels of NLRP3 protein, and NGSDMD. Data are presented as mean ± SD. Significance between groups is indicated by pairwise comparisons. (Control and BHB groups: *n* = 6, cColitis group: *n* = 15, cColitis/BHB & cColitis/KD group: *n* = 12). Statistical analysis was performed by one-way ANOVA followed by Tukey’s *post hoc* test. *p < 0.05, **p < 0.01, ****p < 0.001, ns = no significance.

To further investigate and confirm the development of colonic inflammation following DSS treatment, our study examined the levels of specific parameters including ASC mRNA, NLRP3 mRNA, NLRP3, and NGSDMD. The results, as depicted in Figures 8A, B, Figures 9A, B respectively, revealed a significant increase in these parameters compared to the normal animals. Additionally, The cColitis/KD group exhibited a significant reduction in the levels of NLRP3 mRNA, NLRP3, and NGSDMD compared to the cColitis group. However, no significant change in the levels of ASC mRNA was observed in comparison to the cColitis group. In contrast, the cColitis/BHB group demonstrated a significant decrease in the levels of ASC mRNA, NLRP3 mRNA, NLRP3, and NGSDMD compared to the cColitis group. Furthermore, the administration of exogenous BHB showed superior efficacy over the KD in attenuating the levels of NLRP3 mRNA, NLRP3, and NGSDMD.

### 3.9 Effects of exogenous BHB and KD on BECN1 and P62 levels

**FIGURE 10:**
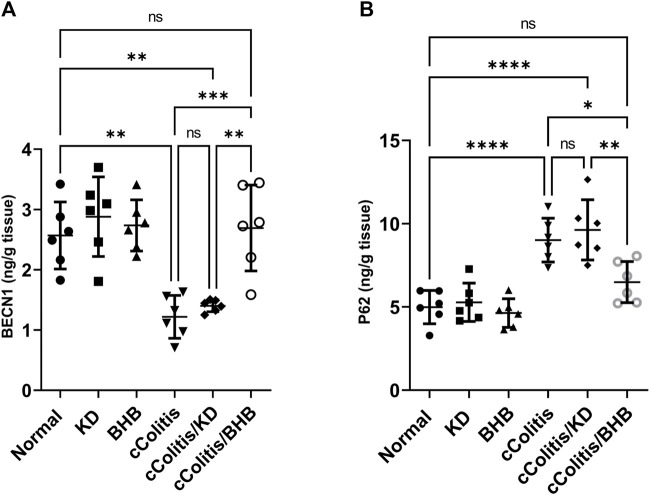
Effects of exogenous BHB and KD on BECN1 **(A)** and P62 **(B)** levels. These findings indicate that DSS-induced colitis is associated with alterations in BECN1 and p62 levels, which are indicative of impaired autophagy. While the KD alone did not correct these alterations, co-administration of BHB with DSS effectively restored BECN1 and p62 levels, suggesting the role of BHB in inducing autophagy. Data are presented as mean ± SD. Significance between groups is indicated by pairwise comparisons. (Control and BHB groups: *n* = 6, cColitis group: *n* = 15, cColitis/BHB & cColitis/KD group: *n* = 12). Statistical analysis was performed by one-way ANOVA followed by Tukey’s *post hoc* test. *p < 0.05, **p < 0.01, ***p < 0.005, ****p < 0.001, ns = no significance.

The administration of DSS resulted in a significant decrease in BECN1 levels compared to the normal group, as illustrated in Figure 10A. Additionally, the results of the cColitis/KD group showed no significant change compared to the cColitis group, indicating that the KD was unable to correct the observed decrease in BECN1. However, the co-administration of BHB with DSS effectively restored the BECN1 levels, highlighting the ability of BHB treatment to induce autophagy. Conversely, DSS led to a significant increase in p62 levels compared to the normal group, as depicted in Figure 10B. Moreover, the results of the cColitis/KD group showed no significant change compared to the cColitis group, confirming the inability of the KD to correct the observed increase in p62. In contrast, the co-administration of BHB with DSS effectively normalized the p62 levels, further confirming the ability of BHB treatment to induce autophagy.

### 3.10 Effects of exogenous BHB and KD on the tight junction proteins, namely, ZO-1, OCLN, and CLDN5 levels

**FIGURE 11:**
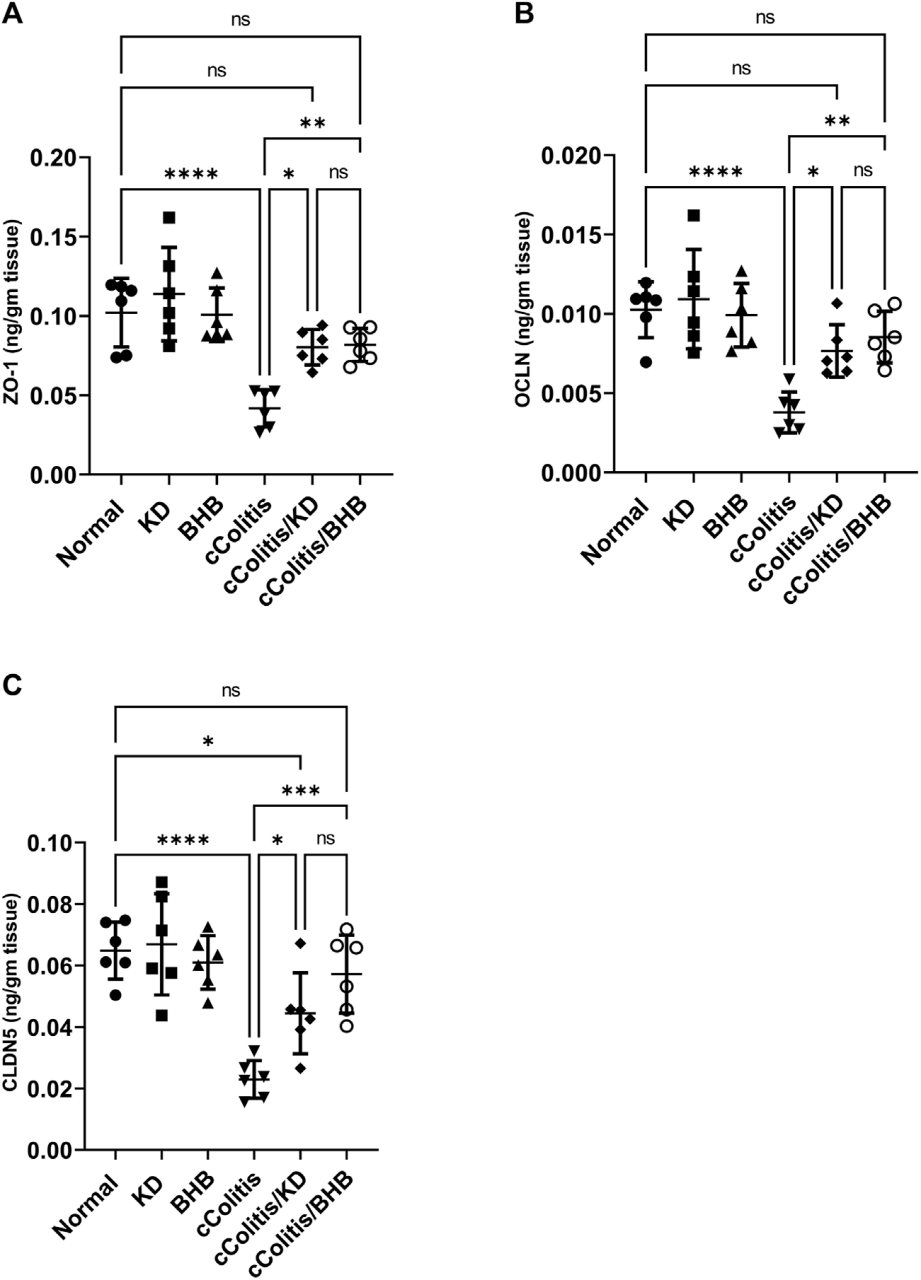
Effects of exogenous BHB and KD on the tight junction proteins, namely, ZO-1 **(A)**, OCLN **(B)**, and CLDN5 **(C)** levels. These findings suggest that DSS-induced colitis leads to a disruption of tight junction proteins, indicating a compromised intestinal barrier. However, both BHB administration and KD feeding effectively restore the levels of these tight junction proteins, indicating their potential in mitigating the DSS-induced leaky gut. Data are presented as mean ± SD. Significance between groups is indicated by pairwise comparisons. (Control and BHB groups: *n* = 6, cColitis group: *n* = 15, cColitis/BHB & cColitis/KD group: *n* = 12). Statistical analysis was performed by one-way ANOVA followed by Tukey’s *post hoc* test. *p < 0.05, **p < 0.01, ***p < 0.005, ****p < 0.001, ns = no significance.

Exposure of rats to DSS resulted in significant decreases of the three investigated tight junction proteins ZO-1, OCLN, and CLDN5, when compared to the cColitis group, as demonstrated in Figures 11A–C, respectively. However, the administration of BHB and the feeding of a KD to DSS-exposed rats successfully restored the decreased levels observed in the cColitis group. This highlights the beneficial role of both BHB and KD in modulating the DSS-induced disruption of the intestinal barrier (leaky gut).

### 3.11 Effects of exogenous BHB and KD on intestinal microbiome composition

**FIGURE 12:**
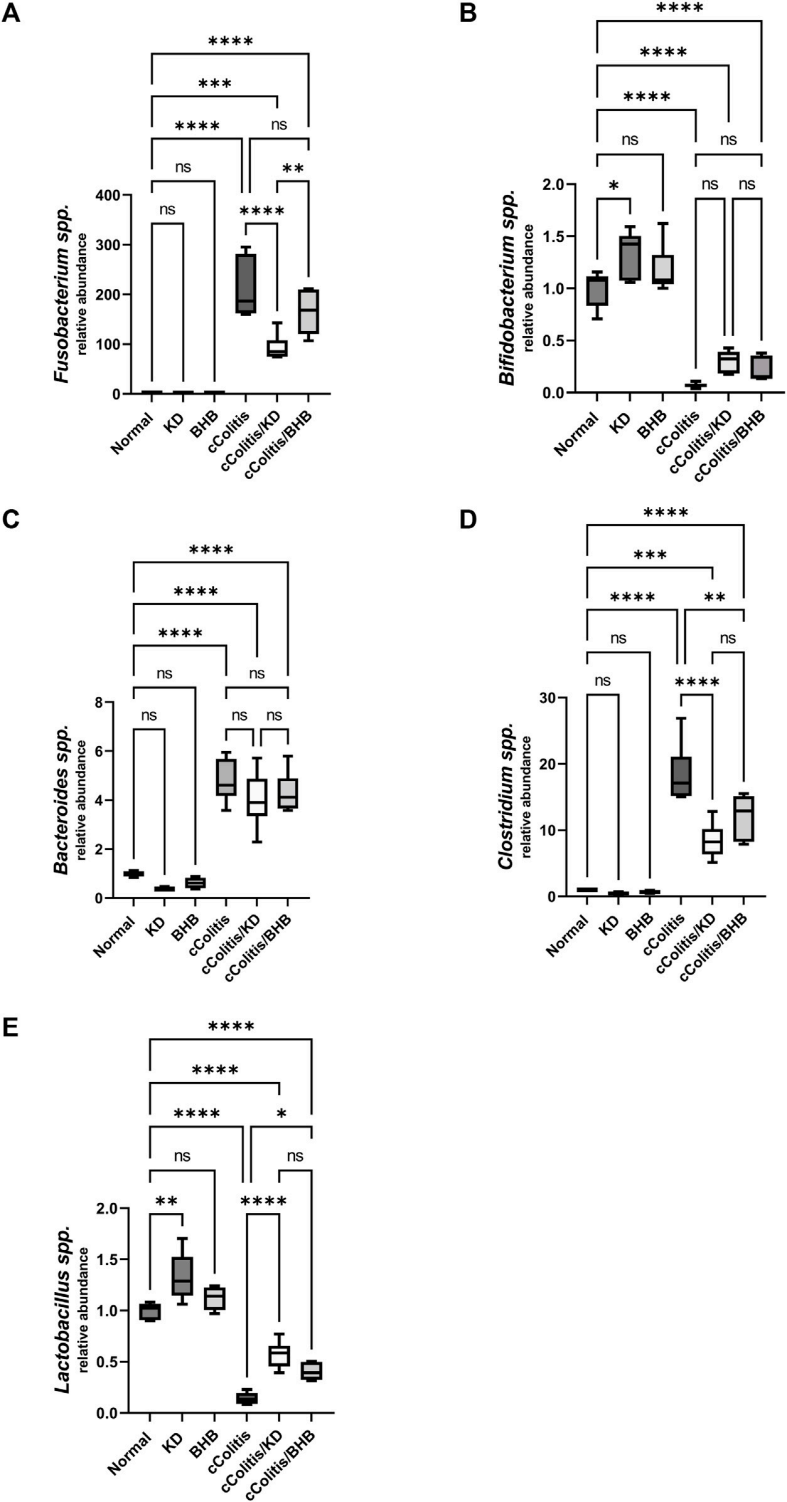
Effects of exogenous BHB and KD on intestinal microbiota. *Fusobacterium* spp. **(A)**, *Bifidobacterium* spp. **(B)**, *Bacteroides* spp. **(C)**, *Clostridium* spp. **(D)**, *Lactobacillus* spp. **(E)**. These findings suggest that DSS treatment leads to alterations in the relative abundance of specific bacterial species in the gut microbiota. While BHB had limited impact, the KD demonstrated significant changes in certain bacterial species. Data are presented as mean ± SD. Significance between groups is indicated by pairwise comparisons. (Control and BHB groups: *n* = 6, cColitis group: *n* = 15, cColitis/BHB & cColitis/KD group: *n* = 12). Statistical analysis was performed by one-way ANOVA followed by Tukey’s *post hoc* test. *p < 0.05,**p < 0.01, ***p < 0.005, ****p < 0.001, ns = no significance.

The relative abundance of each bacterial species in the gut microbiota was assessed where results of current work highlighted that DSS treatment induced a significant increase in the relative abundance of three of the examined bacterial species (*Fusobacterium* spp., *Bacteroides* spp. and *Clostridium* spp., Figures 12A, C, D, respectively), meanwhile, both *Lactobacillus* spp. and *Bifidobacterium* spp. (Figures 12B, E) demonstrated a remarkable suppression following DSS treatment. Interestingly, exogenous BHB had no significant impact on the relative abundance of *F.* spp., *B.* spp., and *B.* spp. and demonstrated a significanr change in *C.* spp., and *L.* spp. compared to the DSS-exposed rat group (cColitis group). However, the KD exhibited a significant change in *F.* spp., *C.* spp., and *L.* spp. and had no significant impact on *B.* spp. and *B.* spp. compared to the cColitis group.

## 4 Discussion

Ulcerative colitis (UC) is a chronic gastrointestinal disorder characterized by recurrent inflammation and ulceration in the colon and/or rectum. It is accompanied by impaired integrity of the intestinal barrier and dysbiosis in the gut microbiota (Saber et al., 2021b; Shaaban et al., 2022). In this article, we demonstrate the beneficial role of BHB administration and a KD in modulating chronic colitis induced by DSS. We found that BHB administration and consuming a KD effectively suppressed the NLRP3 inflammasome signaling, including the priming signal mediated by NFκB. Moreover, we observed a restoration of redox homeostasis in the colon, as evidenced by a decrease in ROS and MDA levels, along with a significant increase in GSH and SOD levels. Significantly, the administration of BHB or following a KD resulted in a decrease in the elevated levels of inflammatory cytokines, such as TNF-α and IL-6, likely due to the inactivation of NFκB. Additionally, the inhibition of NLRP3 led to the downregulation of the active forms of IL-1β and IL-18, due to the repression of caspase-1 activity in conjunction with NFκB inhibition. Moreover, both BHB and KD regimens exhibited the ability to attenuate caspase-3 activation, indicating their antiapoptotic potential.

Indeed, the current data suggest that both BHB and KD have the potential to modulate UC by exerting a protective effect on intestinal barrier function. This protection is evident from the observed induction of three tight junction proteins: ZO-1, OCLN, and CLDN5, which play crucial roles in maintaining the integrity of the intestinal barrier. By promoting the expression of these proteins, BHB and KD may enhance the barrier function and reduce intestinal permeability and protect from leaky gut. The observed effect of BHB on tight junction proteins may be attributed to the BHB-mediated inhibition of the NFκB/NLRP3 signals and the resultant antiapoptotic and anti-pyroptotic potential. This observation aligns well with a previous study that documented the enhanced expression of tight junction proteins, including ZO-1, OCLN, and CLDN5, following caloric restriction accompanied by elevated BHB blood levels. The improvement in tight junctions in that study was shown to improve the integrity of the blood-brain barrier in a mouse glioma model (Jiang and Wang, 2013).

Furthermore, current findings also suggest that both KD and BHB, in part, may contribute to the restoration of the dysbiotic gut microbiota. Imbalances in the gut microbiota have been implicated in the pathogenesis of UC, and the ability of KD and/or BHB to restore microbial composition could contribute to its beneficial effects in UC management. The exact mechanisms underlying this restoration of gut microbiota warrant further investigation. The demonstrated alteration of gut microbiota profile in DSS-induced UC affirmed its putative implication in the disease pathogenesis as stated by previous *in-vivo* studies (Lin et al., 2023). The present study revealed a notable decrease in the levels of Bifidobacterium and *Lactobacillus*, alongside an increase in the abundance of Fusobacteria, *Bacteroides*, and *Clostridium*, following DSS treatment. Furthermore, the supplementation of KD and/or BHB partially mitigated the observed dysbiosis. This was evidenced by a significant increase in the abundance of *Lactobacillus*, and a reduction in the abundance *Clostridium* as reported for both treatment modalities. As per *Fusobacterium*, both KD and BHB induced a decrease in its level however, the reported change was statistically significant for the former and insignificant for the latter. Clearly, the reported difference between the two therapeutic modalities is reasonably acceptable because animals of the two expermintal groups have received totally different diet during the experiment and diet has great impact on the composition of gut microbiome (Trakman et al., 2022a).

These findings align with a previous study that demonstrated how turmeric supplementation improved DSS-induced ulcerative colitis by modulating dysbiosis and promoting the growth of beneficial bacteria (probiotics) (Yang et al., 2022). Moreover, the findings of the current research are in agreement with previous research documented that the administration of probiotics including Bifidobacteria and Lactobacilli resulted in restoring the intestinal microbiota homeostasis in IBD patients alongside enhanced intestinal barrier function (Toscano et al., 2017; Hou et al., 2020). In addition, our results accord well with a recent study by Ni et al. (2023), that documented the mitigation of IBD via the modulation of imbalance in gut microbiota followed by improving intestinal barrier function that was achieved by *Lactobacillus* and Bifidobacterium treatment.

The current research findings indicated that exogenous BHB showed better efficacy in reducing colon inflammation compared to KD. Interestingly, despite its beneficial effect on inflammation, BHB did not significantly impact the composition of the microbiome. This suggests that correcting dysbiosis may not play a significant role in the coloprotective effect of BHB. In addition, KD could not utterly reverse the reported microbiota dysbiosis to completely restore their homeostasis in the present study. Hence, these findings warrant more microbiological investigations.

Intriguingly, our work suggested that the administration of exogenous BHB, as opposed to following a KD, induced autophagic flux as evidenced by the upregulation of BECN1 and the downregulation of p62. Remarkably, p62 is an autophagy adaptor protein that is implicated in selective autophagy through ubiquitnating protein aggregates then transporting them to the autophagosomes (Komatsu et al., 2007; Jiang et al., 2015). Conspicuously, the aggregation of p62 was reported as a clear indicator of impaired autophagy machinery with subsequent accumulation of damaged cellular organelles that could eventually cause cell death.

These findings are noteworthy as they suggest that the therapeutic benefit of BHB is significantly greater than that of KD, possibly due to its ability to induce autophagy. Moreover, this suggests that autophagy induction may occur at higher plasma levels of BHB that are not achieved by the KD alone following our protocol. However, further investigations are required to validate this initial hypothesis that will be conducted in the future work.

Earlier literature has emphasized that the induction of autophagy could potentially play a role in mitigating intestinal inflammation in both Crohn’s disease and UC (Larabi et al., 2020). Indeed, autophagy has been recognized as a critical defense mechanism that plays a pivotal role in the maintenance of intestinal barrier function (Foerster et al., 2022). Additionally, previous studies have provided insights into the role of impaired autophagic activity in the aberrant accumulation of ROS and damaged mitochondrial fragments (Ravindran et al., 2016). Notably, autophagy plays a central role in the ability of cells to discard ROS (Mizushima, 2018). Therefore, the enhancement of autophagy by BHB could provide an explanation, at least partially, for the observed reduction in ROS following BHB administration (Rich et al., 2003).

It is noteworthy that the NLRP3 inflammasome is an intracellular multi-protein complex that plays a central role in innate immunity and inflammation. Its activation leads to the activation of caspase-1, which in turn triggers the maturation of inflammatory cytokines such as IL-1β and IL-18. Previous studies have indicated a positive association between aberrant NLRP3 inflammasome activation and the pathogenesis of UC, making it a potential therapeutic target. The results of the current article support this observation, as we demonstrated that the administration of DSS led to an increase in both NLRP3 mRNA and protein expressions, along with elevated levels of downstream effectors including caspase-1 and its consequences of active IL-1β, and IL-18. Importantly, these discrepancies were effectively attenuated by the administration of BHB and, to a lesser extent, by following a KD. These findings suggest that BHB treatment may lead to the reduction of DSS-induced colonic mucosal injury. Interestingly, our data align with previous research demonstrating the ability of BHB to suppress aberrant NLRP3 inflammasome activation. These findings provide further support for the therapeutic potential of BHB in modulating the inflammatory response associated with UC, particularly by targeting the NLRP3 inflammasome pathway (Neudorf et al., 2020). In consistency with our results, Ning et al. (2023) documented the involvement of NLRP3 suppression with consequent caspase-1 inactivation and decreased IL-1β expression in alleviating DSS-induced UC either *in-vivo* or *in-vitro*.

Initially, it was hypothesized that the capacity of BHB to ameliorate NLRP3 inflammasome activation could be mediated through inhibition of TNF-α as explained by Ning et al. (2023). Another hypothesis that can be proposed to explain the inhibitory effect of exogenous BHB on NLRP3 inflammasome activation is the restriction of ASC mRNA expression, as reported in our current research. This finding aligns with previous studies suggesting that the repression of the ASC scaffold protein oligomer assembly can interrupt both NLRP3 activation and subsequent cytokine secretion, leading to the alleviation of UC symptoms. Notably, ASC is an integral component of the NLRP3 inflammasome and plays a central role in its oligomerization and activation. Mainly ASC bridges the sensor and effector proteins of NLRP3 inflammasome complex enabling the assembly of ternary inflammasome structure. This step occurs following inflammasome activation where NLRP3 firstly recruits ASC adaptor protein followed by pyrin domain (PYD)/PYD interactions between sensor and ASC domains. Secondly, caspase-1 activation would be conducted through caspase activation and recruitment domain (CARD)/CARD interaction between ASC and effector domains (Lu et al., 2014). Thus, the suppression of ASC expression could potentially be responsible for the observed inhibition of NLRP3 inflammasome activation. It is worth noting that our findings are consistent with a previous study that acknowledged the anti-inflammatory effects of BHB, attributing them to the inhibition of NLRP3 inflammasome activation via the inhibition of ASC oligomerization. This provides further support for the notion that BHB can modulate the inflammatory response associated with UC through its influence on the NLRP3 inflammasome pathway (Youm et al., 2015). However, in the current research, it was observed that the KD did not have an impact on the levels of ASC mRNA expression. This finding suggests that the KD-mediated inhibition of the NLRP3 inflammasome is likely independent of ASC, weakening the previously proposed hypotheses. This discrepancy can be explained based on the plasma BHB levels boosted by the KD compared to that exogenously administered. Hence the level of ketosis may have a role.

Generally, findings of the current study aligns very well with previous researches stating the mitigation of experimental colitis and suppression of mucosal inflammation through inhibition of NLRP3 inflammasome activation (Zhou et al., 2017).

Pyroptosis is an inflammatory form of programmed cell death that is mediated by inflammasome activation including NGSDMD, the N-terminal fragment of GSDMD that was reported as the best executioner of pyroptosis (Zheng and Li, 2020). Interestingly, BHB demonstrated a significant capacity to restrain NLRP3/NGSDMD-mediated pyroptosis as evidenced by the reported decreased NGSDMD protein levels and subsequent inhibition of proinflammatory mediators’ expression (e.g., IL-1β and IL-18). Previous reports stated that NGSDMD triggers lytic cell death and the release of several inflammatory mediators that could be implicated in the occurrence and development of colitis. The ability of BHB to suppress pyroptosis via inhibiting the STAT3/NLRP3/GSDMD axis was previously documented in Parkinson’s disease models (Jiang et al., 2022). Additionally, BHB demonstrated a significant capacity to decrease caspase-1 activity and suppress pyroptosis in the renal ischemia-reperfusion injury model (Tajima et al., 2019). Therefore, inhibition of NLRP3/GSDMD-mediated pyroptosis achieved by BHB treatment could establish a novel therapeutic platform for the management of UC.

Another possible explanation for the observed attenuation of UC through the administration of BHB is the restoration of redox homeostasis. This was supported by the significant reduction in ROS and MDA levels, as well as the notable increase in SOD and GSH levels. These findings are consistent with a previous study that demonstrated the ability of the KD to modulate neuroinflammation by increasing circulating levels of BHB, which subsequently activated the antioxidant system and decreased ROS production (Peng et al., 2022). Furthermore, our findings align with a previous article that highlighted the antioxidative effects of BHB. These effects were attributed to the inhibition of class I histone deacetylases (HDACs) by BHB, leading to the activation of Forkhead-box protein O3a (FOXO3a). FOXO3a is known for its role in promoting ROS detoxification and maintaining redox balance within cells. Moreover, FOXO3a was reported to activate gene expression of a range of antioxidants includind catalase, superoxide dismutase and glutathione peroxidase thus contributes to stress resistance, cellular adaptation and survival (Bernardo et al., 2023). This provides further support for the notion that the attenuation of UC observed in our study could be attributed to BHB’s ability to regulate oxidative stress (Shimazu et al., 2013).

In addition to its role in ROS deteoxification, FOXO3a is a transcriptional regulator that could be involved in regulating intestinal inflammation through interfering with nuclear translocation and activation of NFκB with consequent diminished TNF-α production alongside enhanced secretion of the antiniflammatory mediator IL-10. It has been shown that decreases in the production of proinflammatory mediators are associated with NFκB inactivation (Abdelhamid et al., 2022a). Moreover, BHB-mediated FOXO3a activation could potentially contribute to the reported NLRP3 inhbition via suppressed NFκB activation (van Grevenynghe et al., 2012; Yin et al., 2020). This hypothesis will be clearly investigated in our future work shedding more light on BHB-mediated FOXO3a activation as a potential therapeutic target for UC.

Additionally, current work accords well with a previous investigation that highlighted the beneficial role of KD in modulating colitis through decreased lymphoid cell (ILC3) production, mainitaining intestinal barrier function and modifying the intestinal microbiota (Kong et al., 2021). Furthermore, in line with this observation, a recent study in 2022 stated that KD exerted anti-inflammatory effect against IBD in paediatrics and this was potentially attributed to KD impact on gut microbiota as it enhanced the growth of bacterial strains producing short chain fatty acids (Alsharairi, 2022).

However, these observations contradict two earlier studies showed that KD worsened UC through impairment of mucosal barrier function and altering gut microbioal community favouring the growth of pathogenic bacteria (Li et al., 2021; Trakman et al., 2022b). Therefore, it could be concluded that the role of KD in UC is still not clear and requires further clarification and investigation. Moreover, such discrepancy could be attributed to the variability in the composition of gut microbiome. Significantly, The composition of gut microbiota is affected by a myriad of factors such as host genetics, infection, stress and geography, whilst diet is considered the strongest influence (Quigley, 2017; Cabrera-Mulero et al., 2019).

**FIGURE 13:**
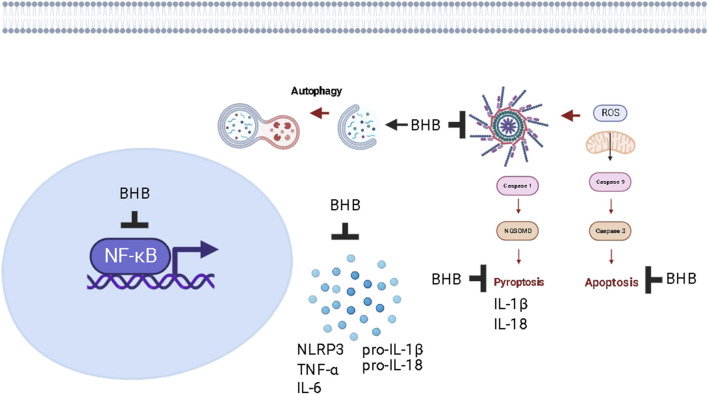
The proposed mechanism of action of increasing plasma levels of BHB as a protective strategy against chronic colitis in rats.

The current study provides valuable insights into the beneficial effects of exogenous BHB and KD consumption in alleviating DSS-induced UC. Both regimens show promise in improving micro and macrostructures of the inflamed colon, preventing DSS-induced weight loss, and attenuating disease activity. However, BHB was demonstrated to be superior to KD in various aspects. Mechanistically, the study showed that these therapeutic interventions exert anti-inflammatory effects, potentially attributed to the inhibition of the aberrantly activated NFκB/NLRP3 signaling pathway. This was evidenced by the improvement in levels of downstream inflammatory mediators such as TNF-α, IL-6, IL-1β, and IL-18, along with the inactivation of caspase-3, indicating antiapoptotic activity. The study also revealed the downregulation of NGSDMD, suggesting anti-pyroptotic potential (Figure 13). Furthermore, KD partially corrected the imbalance in the intestinal microbiota. Both interventions contributed to the modulation of intestinal barrier disruption and improved intestinal barrier function. However, exogenous BHB exhibited autophagy induction capabilities, which may explain its superiority over KD in alleviating colitis. This also implies that the coloprotective effect of BHB may be dependent on its plasma concentration. Overall, the current study provides promising initial evidence regarding the pharmacological benefits of BHB in mitigating DSS-induced UC. However, further research is necessary to fully explore the potential of BHB or BHB-boosting compounds as therapeutic interventions for UC.

Future studies should aim to investigate the specific molecular mechanisms through which a BHB-boosting molecule modulates autophagy and corrects dysbiosis in the context of UC. Understanding these mechanisms in greater detail will provide insights into the targets involved, potentially leading to the development of more targeted and effective therapies. Additionally, it would be valuable to explore the therapeutic potential of BHB in other models of UC and in clinical trials involving human subjects. This would provide a more comprehensive understanding of its efficacy, safety, and optimal dosing regimens for UC treatment. In conclusion, while the current study presents promising findings, further research is warranted to fully explore the potential of BHB or BHB-boosting compounds as therapeutic interventions for UC and to unravel the underlying molecular mechanisms involved in autophagy modulation and dysbiosis correction. Additionally, elucidating autophagic processes through techniques like electron microscopy and immunohistochemical labeling of autophagic proteins, such as LC3, ULK, and ATG proteins. Similarly, TUNEL assay would be of value in elucidating apoptosis.

## Data Availability

The original contributions presented in the study are included in the article/supplementary material, further inquiries can be directed to the corresponding authors.

## Abbreviations

ASC, apoptosis-associated speck-like protein containing a CARD; BECN1, Beclin-1; BHB, β-hydroxybutyrate; cColitis, chronic colitis; CLDN5, claudin-5; DAI, disease activity score; DSS, dextran sodium sulfate; FOXO3a, forkhead-box protein O3a transcription factor; GIT, gastrointestinal tract; HDACs, class I histone deacetylases; IBD, inflammatory bowel disease; KD, ketogenic diet; MDA, malondialdehyde; MDI, macroscopic damage index; MPO, myeloperoxidase; NFκB, nuclear transcription factor kappa B; NGSDMD, gasdermin D N-terminal fragment; NLRP3, nucleotide-binding oligomerization domain-like receptor protein 3; OCLN, occludin; p62, sequestosome-1; ROS, reactive oxygen species; SOD, superoxide dismutase; TNF-α, tumor necrosis factor-alpha; UC, ulcerative colitis; ZO-1, zonula occludens-1.
