# Corrigendum: Unveiling the therapeutic potential of exogenous β-hydroxybutyrate for chronic colitis in rats: novel insights on autophagy, apoptosis, and pyroptosis

**DOI:** 10.3389/fphar.2024.1382529

**Published:** 2024-03-13

**Authors:** Rasha Abdelhady, Sameh Saber, Mustafa Ahmed Abdel-Reheim, Mohannad Mohammad S. Alamri, Jaber Alfaifi, Masoud I. E. Adam, Lobna A. Saleh, Azza I. Farag, Elsayed A. Elmorsy, Hend S. El-Wakeel, Ahmed S. Doghish, Mohamed E. Shaker, Sara H. Hazem, Heba A. Ramadan, Rabab S. Hamad, Osama A. Mohammed

**Affiliations:** ^1^ Pharmacology and Toxicology Department, Faculty of Pharmacy, Fayoum University, Fayoum, Egypt; ^2^ Department of Pharmacology, Faculty of Pharmacy, Delta University for Science and Technology, Gamasa, Egypt; ^3^ Department of Pharmaceutical Sciences, College of Pharmacy, Shaqra University, Shaqra, Saudi Arabia; ^4^ Department of Pharmacology and Toxicology, Faculty of Pharmacy, Beni-Suef University, Beni Suef, Egypt; ^5^ Department of Family Medicine, College of Medicine, University of Bisha, Bisha, Saudi Arabia; ^6^ Department of Child Health, College of Medicine, University of Bisha, Bisha, Saudi Arabia; ^7^ Department of Medical Education and Internal Medicine, College of Medicine, University of Bisha, Bisha, Saudi Arabia; ^8^ Department of Clinical Pharmacology, Faculty of Medicine, Ain Shams University, Cairo, Egypt; ^9^ Department of Pharmacology and Toxicology, Collage of Pharmacy, Taif University, Taif, Saudi Arabia; ^10^ Department of Human Anatomy and Embryology, Faculty of Medicine, Zagazig University, Zagazig, Egypt; ^11^ Department of Pharmacology and Therapeutics, Qassim College of Medicine, Qassim University, Buraydah, Saudi Arabia; ^12^ Department of Clinical Pharmacology, Faculty of Medicine, Mansoura University, Mansoura, Egypt; ^13^ Physiology Department, Benha Faculty of Medicine, Benha University, Banha, Egypt; ^14^ Physiology Department, Al-baha Faculty of Medicine, Al-baha University, AlBaha, Saudi Arabia; ^15^ Department of Biochemistry, Faculty of Pharmacy, Badr University in Cairo (BUC), Cairo, Egypt; ^16^ Department of Biochemistry and Molecular Biology, Faculty of Pharmacy (Boys), Al-Azhar University, Cairo, Egypt; ^17^ Department of Pharmacology, College of Pharmacy, Jouf University, Sakaka, Saudi Arabia; ^18^ Department of Pharmacology and Toxicology, Faculty of Pharmacy, Mansoura University, Mansoura, Egypt; ^19^ Department of Microbiology and Immunology, Faculty of Pharmacy, Delta University for Science and Technology, Al Mansurah, Egypt; ^20^ Biological Sciences Department, College of Science, King Faisal University, Al Ahsa, Saudi Arabia; ^21^ Central Laboratory, Theodor Bilharz Research Institute, Giza, Egypt; ^22^ Department of Clinical Pharmacology, College of Medicine, University of Bisha, Bisha, Saudi Arabia

**Keywords:** ulcerative colitis, β-hydroxybutyrate, NLRP3 inflammasome, apoptosis, pyroptosis

In the published article, there was an error in affiliations 11 and 12. Instead of:

“^11^Department of Clinical Pharmacology, Faculty of Medicine, Mansoura University, Mansoura, Egypt.”

“^12^Pharmacology and Therapeutics Department, Qassim College of Medicine, Qassim University, Buraydah, Saudi Arabia.”

it should be:

“^11^Department of Pharmacology and Therapeutics, Qassim College of Medicine, Qassim University, Buraydah, Saudi Arabia.”

“^12^Department of Clinical Pharmacology, Faculty of Medicine, Mansoura University, Mansoura, Egypt.”

The authors apologize for this error and state that this does not change the scientific conclusions of the article in any way. The original article has been updated.

